# Protein fibers with self-recoverable mechanical properties via dynamic imine chemistry

**DOI:** 10.1038/s41467-023-41084-1

**Published:** 2023-09-02

**Authors:** Jing Sun, Haonan He, Kelu Zhao, Wenhao Cheng, Yuanxin Li, Peng Zhang, Sikang Wan, Yawei Liu, Mengyao Wang, Ming Li, Zheng Wei, Bo Li, Yi Zhang, Cong Li, Yao Sun, Jianlei Shen, Jingjing Li, Fan Wang, Chao Ma, Yang Tian, Juanjuan Su, Dong Chen, Chunhai Fan, Hongjie Zhang, Kai Liu

**Affiliations:** 1 School of Chemistry and Molecular Engineering, Shanghai Engineering Research Center of Molecular Therapeutics and New Drug Development, East China Normal University, Shanghai, 200241 China; 2 Engineering Research Center of Advanced Rare Earth Materials (Ministry of Education), Department of Chemistry, Tsinghua University, 100084 Beijing, China; 3 State Key Laboratory of Rare Earth Resource Utilization, Changchun Institute of Applied Chemistry, Chinese Academy of Sciences, 130022 Changchun, China; 4 Frontiers Science Center for Transformative Molecules, School of Chemistry and Chemical Engineering, and Institute of Molecular Medicine, Renji Hospital, School of Medicine, Shanghai Jiao Tong University, Shanghai, 200240 China; 5 Center of Materials Science and Optoelectronics Engineering, College of Materials Science and Optoelectronic Technology, University of Chinese Academy of Sciences, Beijing, 100049 China; 6 College of Energy Engineering, Zhejiang University, Hangzhou, 310027 China

**Keywords:** Mechanical properties, Bioinspired materials, Biopolymers

## Abstract

The manipulation of internal interactions at the molecular level within biological fibers is of particular importance but challenging, severely limiting their tunability in macroscopic performances and applications. It thus becomes imperative to explore new approaches to enhance biological fibers’ stability and environmental tolerance and to impart them with diverse functionalities, such as mechanical recoverability and stimulus-triggered responses. Herein, we develop a dynamic imine fiber chemistry (DIFC) approach to engineer molecular interactions to fabricate strong and tough protein fibers with recoverability and actuating behaviors. The resulting DIF fibers exhibit extraordinary mechanical performances, outperforming many recombinant silks and synthetic polymer fibers. Remarkably, impaired DIF fibers caused by fatigue or strong acid treatment are quickly recovered in water directed by the DIFC strategy. Reproducible mechanical performance is thus observed. The DIF fibers also exhibit exotic mechanical stability at extreme temperatures (e.g., −196 °C and 150 °C). When triggered by humidity, the DIFC endows the protein fibers with diverse actuation behaviors, such as self-folding, self-stretching, and self-contracting. Therefore, the established DIFC represents an alternative strategy to strengthen biological fibers and may pave the way for their high-tech applications.

## Abstract (web summary)

Manipulating the internal interactions within biological fibers to tune macroscopic properties is important but challenging, and can limit applications. Here, the authors report the use of dynamic imine chemistry for engineering molecular interactions and forming strong and tough protein fibers.

## Introduction

High-performance protein fibers such as spider silks have attracted substantial attention owing to their mechanical properties^1^ and good biocompatibility^2^. In such systems, supramolecular interactions such as hydrogen bonding are involved in the formation of crystalline and amorphous structures, which are crucial for the manipulation of fibers’ diverse properties^3^. The labile nature of supramolecular interactions^4,5^, however, leads to undesirable restrictions in the tunability of fibers’ structures and functions. In addition, protein fibers dominated by supramolecular structures often lack mechanical adaptability to extreme conditions, under which their mechanical performances would be severely compromised and challenging to recover^6^. Regenerated silk fibers, for instance, cannot fully reconstruct their β-sheet structures after regeneration treatment with strong polar solvents, weakening mechanical strength^7^. Consequently, it is urgent to develop new chemical strategies to engineer protein fibers with mechanical maneuverability, recoverability, and environmental tolerance.

The robust yet reversible dynamic covalent bonds show great potential in the rational design of smart materials where they can be broken and reformed^8^. This opens up new prospects to functionalize materials with unprecedented structural and dynamic characteristics, exhibiting a macroscopic response to an external stimulus^9^. Dynamic covalent bonds, such as imine bonds^10^, boronic ester bonds^11^, and disulfide bonds^12^, exist almost exclusively in polymer materials, e.g., hydrogels, offering them remarkable macroscopic responses, such as self-healing and malleability^13^. Although numerous efforts have been devoted to achieving tunable mechanical performances using dynamic covalent bonds, the materials exhibited relatively weak mechanical strength ranging from several kilopascals to tens of megapascals^14^. It would be highly desirable to develop new dynamic bonding systems that can achieve ultra-strong mechanical performance. In particular, recent advances in the fabrication of robust structural protein fibers have shown diverse applications^15–17^; however, the modulation of their dynamic mechanical performances and malleability at the macroscopic level is still elusive. Therefore, the introduction of dynamic bonding interactions into protein fibers for the realization of desired reversible and robust mechanical behaviors might become an attractive goal.

Herein, we introduce the DIFC strategy into the K-protein systems and construct mechanically strong protein fibers with recoverable properties. In stark contrast to conventional protein fibers without environmental adaptability, the DIF fibers exhibited high mechanical strength and toughness, temperature tolerance capacity, as well as remarkable recoverability, outperforming many synthetic protein and polymer fibers. The DIFC endowed the protein fibers with multiple humidity-responsive modes, showing various actuation behaviors, including self-folding, self-stretching, and self-contracting. Therefore, this work might promote the DIFC as a methodology for the fabrication of high-performance biological fibers.

## Results and discussions

### Design and fabrication of high-performance DIF fibers

**Fig. 1:**
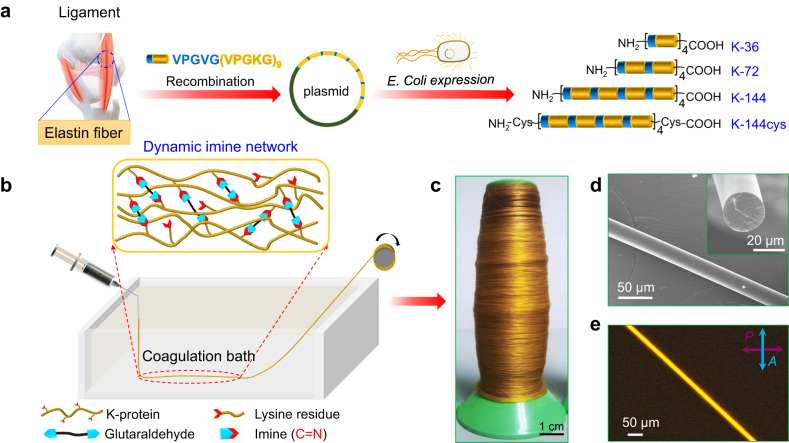
Design and preparation of high-performance DIF fibers by developing dynamic imine fiber chemistry. **a** Biomimetic design and expression of recombinant elastin-like proteins (ELPs). (VPGVG (VPGKG)_9_)_n_ with repeat numbers of *n* = 4, 8, and 16, termed as K-36, K-72, and K-144, respectively, were expressed in *E. coli*. Cysteine residues were introduced to the N- and C-terminals of K-144, forming K-144cys. **b** Schematics showing the preparation of DIF fibers by wet spinning. K-protein solution was squeezed into a coagulation bath with 1% glutaraldehyde (GA), in which GA molecules rapidly react with lysine residues on the protein chains, forming dynamic imine cross-linking networks. The DIF fibers were then collected on a rotor. **c** Photograph of the as-spun DIF-72 fiber, showing a golden color. **d** Scanning electron microscopy (SEM) image of a post-stretched DIF-72 fiber shows a uniform diameter of ~25 μm and a smooth surface. The inset shows the cross-section of the fiber. **e** Polarized optical microscopy (POM) image of a post-stretched DIF-72 fiber shows a high birefringence, indicating an orientationally ordered arrangement of proteins within the fiber. Three parallel measurements were performed for each SEM and POM analysis.

Elastin-like protein is a biomimetic recombinant protein with repeat units (VPGVG)_m_(VPGXG)_n_, where X is an amino acid other than proline. Herein, lysine is incorporated to provide covalent reaction sites, where amine groups can react with aldehydes to form dynamic imine networks. The design and fabrication of high-performance protein fibers regulated by DIFC are illustrated in Fig. 1. Biomimetically designed recombinant ELPs, VPGVG(VPGKG)_n_, from the mammalian ligament^18,19^ were successfully expressed in *Escherichia coli* (*E. coli*) cell factory and purified by liquid chromatography^20,21^. Considering the high efficiency of plasmid construction of basic repeating unit VPGVG(VPGKG)_9_, the initial monomer gene was designed as K-9, and subsequently synthesized protein sequences are all integer multiples of K-9 in length (Supplementary Table 1). K-36, K-72, and K-144 were designed for the DIF fibers study. In addition, K-144 protein functionalized with cysteine residues at both ends is termed K-144cys, which aims to further increase the protein’s molecular weight via cross-linking through disulfide bonds (S-S). Because K-proteins contain many positively charged lysine moieties, which greatly increase their hydrophilicity and cause the disappearance of phase transition^22^, K-proteins could not be purified by inverse transition cycling^23^. Histidine tag-labeled K-proteins were purified using high-performance liquid chromatography (HPLC) and characterized by sodium dodecyl sulfate-polyacrylamide gel electrophoresis (SDS-PAGE), matrix-assisted laser desorption/ionization time-of-flight (MALDI-TOF) mass spectrometry, proton nuclear magnetic resonance (^1^H NMR), and high-performance liquid chromatography-mass spectrometric (HPLC-MS) (Supplementary Figs. 1–5). Further HPLC analysis confirmed that the successful expression of K-proteins with purity in the range of 84-99% (95.8% for K-36, 99.4% for K-72, 98.2% for K-144, and 88.4% for K-144cys) (Supplementary Fig. 6). Of note, SDS-PAGE analysis revealed the presence of slightly degraded polypeptide fragments in K-72, K-144, and K-144cys. This observation might be attributed to the proteases, especially trypsin and elastase. Despite the use of protease inhibitors in the preparation process, the degradation cannot be completely avoided. In particular, the large amount of positively charged lysine residues in these proteins is the main site of trypsin and elastase^24^, which leads to partial degradation. By optimizing the fermentation parameters of a 100 L fermentor, the final purification yield of K-72 protein is ~400 mg/L (Supplementary Fig. 3 and Supplementary Table 2), which is among the top level of protein yields when compared with other reported work^25–28^. Of note, an obvious dimerization of K-144cys was observed in SDS-PAGE analysis due to the formation of disulfide bonds between two K-144cys chains. A library of DIF fibers was prepared by wet spinning through a coagulation bath with glutaraldehyde (GA), where a dynamic imine network between amino groups in lysine residues and aldehydes in GA was formed via aldehyde-amino condensation (Fig. 1b). During the extrusion process, the shear force was generated by the nozzle with an inner diameter of 210 μm and induced ordering in the protein fibers, facilitating the formation of both covalent and noncovalent interactions and thus leading to a stronger mechanical property^29^. With the optimized spinning conditions, the massive production of golden DIF fibers was achieved (Fig. 1c). It should mention that only 1w/w% GA is involved during cross-linking in the present system. This is much lower than the clinically used content (8–10 w/w% GA) in a commercial biomedical glue (BioGlue®) approved by the FDA or other reported systems^30–32^. Regarding the cytotoxicity of K-proteins, it is worth noting that the recombinant proteins in our study contain the repeat unit of VPGVG(VPGKG)_9_ and positively-charged lysines were arranged at intervals in K-proteins instead of next to each other. Thus, the charge density of our protein is much less than other polycationic proteins, e.g., polylysines. The Cell Counting Kit 8 (CCK-8) assay showed that the cell viability is still above 88% when the concentration of K-protein is as high as 500 μg·mL^−1^, indicating low cytotoxicity of K-proteins (Supplementary Fig. 7). The cytotoxicity of DIF fibers was further investigated by co-culturing fibers with HEK293T, L929, or HT22 cells following the ISO 10993-5: 2009 guidelines (Supplementary Figs. 8–11). After 24 h co-culture, calcein-AM and propidium iodide (PI) staining assays were used to stain live and dead cells, respectively. The results confirmed that the DIF fibers barely exhibited any toxicity to co-cultured cells, even for cells adhered on the fiber surfaces. Such low cytotoxicity of DIF fibers is attributed to the rather low concentration of GA (1 w/w%) used to form the cross-linking network between amino groups of lysine residues and aldehyde. Besides, most positively charged lysines were consumed by cross-linking with GA. In contrast, the reported collagen/PVA film was cross-linked with 8 w/w% GA^31^. Therefore, our DIF fibers exhibited rather low toxicity.

### Characterization of the DIF fibers

To optimize the structural orientation and strengthen the mechanical properties, the as-spun DIF fibers were post-stretched by soaking them with water and drawn to 100% of their original length. The surface morphology and cross-section of the post-stretched DIF fibers investigated by scanning electron microscopy (SEM) revealed that post-stretched DIF-72 samples were uniform in diameter and solid without any noticeable structural defects (Fig. 1d and Supplementary Figs. 12, 13). In addition, the post-stretched DIF fibers showed a significant birefringence under crossed polarized optical microscopy (POM), suggesting well-aligned protein chains within the fibers^33^ (Fig. 1e). Conversely, no birefringence was observed in the as-spun DIF fibers (Supplementary Fig. 14). 2D small-angle X-ray scattering (SAXS) using synchrotron radiation further confirmed the POM observations, in which the post-stretched fibers exhibited two sharp streaks while the as-spun fibers only showed two dispersed signals (Supplementary Figs. 15–18). The periodic ordering distance (**L**) along the radial direction of the fiber was determined through linear fitting of the *B*^2^_obs_ - *q*^−2^ curve of 2D SAXS pattern, with the equation ${{{{{\bf{L}}}}}}={{{{{\bf{2}}}}}}{{{{{\boldsymbol{\pi }}}}}}/\sqrt{{{{{{\boldsymbol{k}}}}}}}$, where ${{{{{\boldsymbol{k}}}}}}$ is the slope of *B*^2^_obs_ - *q*^−2^ curve^34^. It was found that the periodic ordering distance decreases from 34.1 nm, 36.9 nm, 22.9 nm, and 26.1 nm in the as-spun DIF-144cys, DIF-144, DIF-72, and DIF-36 fiber to 16.2 nm, 13.4 nm, 15.2 nm, and 10.1 nm in the post-stretched DIF-144cys, DIF-144, DIF-72, and DIF-36 fiber, respectively. It is noteworthy that the cysteine-induced formation of disulfide bonds does not cause any alternation in the molecular ordering of fibers compared to other DIF fibers, which is confirmed by POM and 2D-SAXS analysis (Supplementary Figs. 14–18). The K-proteins are randomly coiled, as evidenced by circular dichroism analysis (Supplementary Fig. 19). The SAXS analysis shows that (1) the post-stretching treatment is important for the formation of long-range ordered molecular structures and might contribute to strengthened supramolecular interactions within the fibers^35^, guaranteeing remarkable mechanical properties; (2) the different lengths of the K-proteins have a negligible effect on the alignment and conformation of protein molecules within the DIF fibers.

### Mechanical performances of the DIF fibers

**Fig. 2:**
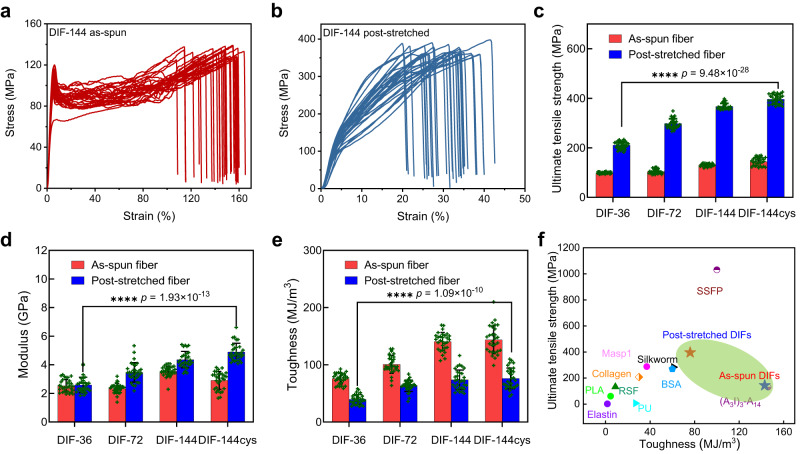
High mechanical performances of the DIF fibers. **a**, **b** Representative Stress-strain curves of the as-spun (**a**) and 100% post-stretched (**b**) DIF-144 fibers. All DIF samples have uniform diameters, which are measured by POM before testing. Generally, the as-spun DIF fibers have a diameter of around 25 µm, while 100% post-stretched DIFs have a diameter of approximately 20 µm. **c**–**e** The statistical ultimate tensile strength (**c**), Young’s modulus (**d**), and toughness (**e**) of as-spun and 100% post-stretched DIFs. The asterisks denote the statistical significance: ****p < 0.0001. All data (**c**–**e**) in the graphs are mean values of standard deviation (mean ± SD, *n* = 30). Statistical analysis was performed with GraphPad Prism 8.0 and Origin 2021 software. The results (**c**–**e**) are obtained by the statistical approach of two-way *t*-tests. **f** Comparison of ultimate tensile strength and toughness between as-spun/post-stretched DIF fibers and other reported fibers produced from elastin^41^, regenerated silk fibroin (RSF)^6^, collagen^42^, bovine serum albumin (BSA)^35^, *S.c. ricini* silkworm^43^, major ampullate silk protein 1 (MaSp1)^44^, engineered mini-spidroins ((A_3_I)_3_-A_14_)^45^, silk-SI-fusion proteins (SSFP)^45^, poly(lactic acid) (PLA)^48^ and polyurethane (PU)^49^.

The mechanical properties of as-spun and post-stretched DIF fibers were systematically evaluated regarding ultimate tensile strength, Young’s modulus, and toughness using a tensile tester under ambient conditions (25 °C, ~30% humidity, and nonsterile) (Fig. 2, Supplementary Figs. 20–22, and Supplementary Table 3). The typical stress-strain curves of the as-spun and post-stretched DIF-144 fibers were illustrated in Fig. 2a, b. The results demonstrated an increase in ultimate tensile strength as the protein molecular weight increased from 19 kDa (K-36) to 72 kDa (K-144cys) (Fig. 2c–e, and Supplementary Figs. 20–22), indicating that protein size plays a significant role in regulating the mechanical strength of the fibers^36,37^. For the as-spun DIF fibers, the mechanical performance of fibers increased as the molecular weight of K-proteins K-36, K-72, K-144, and K-144cys increased. This resulted in an increase in ultimate tensile strengths from 98.9 ± 3.3 MPa to 144.1 ± 16.3 MPa, corresponding toughness from 70.6 ± 9.3 MJ/m^3^ to 143.8 ± 24.5 MJ/m^3^, and the corresponding Young’s moduli from 2.5 ± 0.4 to 2.9 ± 0.6 GPa. It was found that there is an obvious plateau of plastic deformation after the yielding point owing to the disordered orientation of the internal structure of as-spun fibers, resulting in high extensibility and toughness but low ultimate tensile strength. In stark contrast, the post-stretched fibers exhibited distinct mechanical behaviors after post-stretching treatment, yielding much higher ultimate tensile strength in the range of 200~420 MPa and stiffness in the range of 2~5.5 GPa (Fig. 2c, d). This is because the post-stretching treatment is crucial to improve the alignment of protein chains and might decrease the intermolecular defects, thus eliminating the necking and improving the mechanical strength and stiffness^38,39^. However, it should be noted that the toughness of post-stretched DIF fibers was lower than the corresponding as-spun samples (Fig. 2e). This phenomenon has been observed in traditional polymer fibers^38,40^, the decreased polymer chain entanglement within fibers by post-stretching would reduce their extensibility and toughness. It has been found that the post-stretched DIF fibers with varying molecular weights exhibit a similar strain at break. This could be attributed to the fact that the fibers with different molecular weights have the same sequence, and the strain provided by each sequence after cross-linking is basically the same, thus presenting a similar strain at break. The post-stretched DIF-144cys fibers exhibit an ultimate tensile strength and Young’s modulus of 396 ± 18 MPa and 4.9 ± 0.6 GPa, respectively, while their toughness remains above 70 MJ/m^3^, outperforming many artificial synthetic protein^6,35,41–47^ and polymer fibers^48,49^ (Fig. 2f). Notably, although the K-144 and K-144cys proteins have nearly the same molecular weight, the ultimate tensile strength of post-stretched DIF-144cys fibers was approximately 30 MPa higher than that of the post-stretched DIF-144 fiber, which could be attributed to the dimerization between two K-144cys proteins via disulfide bonds, thus forming longer protein chains and showing better mechanical performances. These findings suggest that high molecular weight and well-aligned molecular arrangement induced by post-stretching treatment are essential to improve the mechanical performance of the DIF fibers.

### Mechanical recoverability of the DIF fibers

**Fig. 3:**
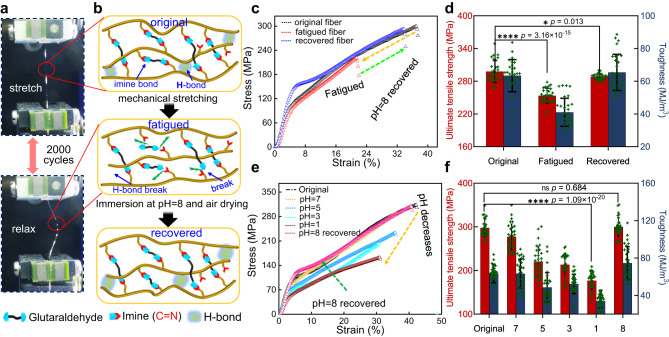
Recoverable mechanical properties of the DIF fibers regulated by dynamic imine network. **a** Fatigue test of the 100% post-stretched DIF-72 fiber as an example. The fiber was alternatively stretched (20% strain) and relaxed for 2000 cycles. **b** Schematics illustrates the underlying mechanism for the recovery of fatigued DIF-72 fiber at pH = 8. Some imine bonds and hydrogen bonds were broken under repeated mechanical stretching, which could be repaired by immersing fibers in a pH = 8 aqueous solution with 50 mm Na_3_PO_4_ followed by air drying. **c** Stress-strain curves of original, fatigued, and recovered DIF-72 fibers. **d** Ultimate tensile strength and toughness of original, fatigued, and recovered DIF-72 fibers. Fatigued fibers essentially restored their mechanical performances after immersion at pH = 8 and re-dried in the air. **e** Stress-strain curves of the DIF-72 fibers immersed at pH = 7, 5, 3, 1 for 12 h and recovered at pH = 8 for 12 h. **f** Ultimate tensile strength and toughness of the post-stretched DIF-72 fibers under different pH treatments. The mechanical performances of post-stretched DIF-72 fibers were weakened as the pH decreased, which was recovered at pH = 8. The asterisks denote the statistical significance: *p < 0.05 and ****p < 0.0001. ns no significant difference. All data (**d**), (**f**) in the graphs are mean values of standard deviation (mean ± SD, *n* = 30). Statistical analysis was performed with GraphPad Prism 8.0 and Origin 2021 software. The results (**d**), (**f**) are obtained by the statistical approach of two-way *t*-tests.

Since imine bonds are dynamically reversible^50^, fractured imine bonds could be regenerated, making it possible to recover and tune the mechanical performances of DIF fibers. All K-proteins have the same fundamental repeat sequence of VPGVG (VPGKG)_9_, and thus the DIF fibers with different molecular weights are expected to possess a similar dynamic imine network and show a similar recoverable performance. To investigate the mechanical recoverability of the DIF fibers, the DIF-72 fiber was selected as a representative example due to the medium molecular weight and high expression yield of K-72 protein. Under cyclic loading and unloading at a small strain of 5%, the ultimate tensile strength of post-stretched DIF-72 fibers remained unchanged and barely affected even after 50 cycles (Supplementary Fig. 23). However, the ultimate tensile strength and toughness of the post-stretched DIF-72 fibers decreased significantly under 20% strain over 10 cyclic loading-unloading loops (Supplementary Fig. 24). After 2000 cycles at 20% strain, the post-stretched DIF-72 fibers became fatigued (Fig. 3a), resulting in reduced mechanical performance. This may be due to the breakage of some imine bonds in the cross-linking network and hydrogen bonds (HBs) between amides during repeated mechanical stretching, as modeled in Fig. 3b. As a result, the ultimate tensile strength, toughness, and extensibility declined to 253.6 ± 13.5 MPa, 39.7 ± 8.3 MJ/m^3^, and 24.1 ± 5.1%, respectively (Fig. 3c). To recover the mechanical performances, the post-stretched DIF-72 fibers were simply immersed into an aqueous solution (pH = 8, 50 mm Na_3_PO_4_) and re-dried in the air, through which the broken imine bonds and HBs were regenerated. As anticipated, the mechanical performances of recovered fibers were essentially restored to their original values (Fig. 3c, Supplementary Fig. 25, and Supplementary Table 4). The ultimate tensile strength, toughness, and extensibility are recovered to 288.2 ± 5.5 MPa, 65.4 ± 11.0 MJ/m^3^, and 33.2 ± 5.2%, respectively (Fig. 3d). All these results showed that the post-stretched DIF fibers have good anti-fatigue feature.

In addition to the anti-fatigue properties, the mechanical performances of DIF-72 fibers demonstrated pH-dependent behaviors owing to the broken and reformed dynamic imine networks (Fig. 3e, Supplementary Figs. 26–29, and Supplementary Table 5). When the as-spun DIF-72 fibers were treated with decreased pH environments, the ultimate tensile strength decreased from 106.7 ± 19.5 MPa (pH = 7) to 88.8 ± 13.9 MPa (pH = 1), while the corresponding toughness values remained in the range of 110 to 120 MJ/m^3^ without significant difference. The decreased ultimate tensile strength of the as-spun DIF-72 fibers was due to the broken imine bonds caused by acid treatment. Of note, the designed K-proteins have a random coil structure. Such structures in the as-spun DIF-72 fibers could retain their intricate entanglement, thereby potentially helping to maintain the fiber’s toughness at a relatively consistent level, even when subjected to different pH environments. Consequently, there is no significant variation in the toughness of the as-spun DIF-72 fibers. Then the pH treated as-spun DIF-72 fibers were immersed into a pH = 8 aqueous solution with 50 mm Na_3_PO_4_, resulting in these values being recovered to their original value due to the reformed dynamic imine bonds (Fig. 3f, and Supplementary Table 5).

Subsequently, the tensile tests showed that the ultimate tensile strength and toughness of post-stretched DIF-72 fibers were significantly decreased from 277.2 ± 29.2 MPa (pH = 7) to 176.7 ± 19.1 MPa (pH = 1) and from 64.2 ± 14.7 MJ/m^3^ (pH = 7) to 34 ± 7.2 MJ/m^3^ (pH = 1). In this process, the imine bonds in the densely packed cross-linking networks were partly broken within the post-stretched DIF-72 fibers after being treated with acid, significantly weakening both ultimate tensile strength and toughness. Similarly, the mechanical performance of post-stretched DIF-72 fibers was recovered when dynamic imine bonds were reformed after immersion in an aqueous solution (pH = 8, 50 mm Na_3_PO_4_) (Supplementary Figs. 26–29). Of note, the variations in pH have minimal impact on the conformation of the K-proteins but the imine bonds could break and reform within the DIF fibers, as confirmed by FT-IR spectroscopy (Supplementary Fig. 30). After being treated with acid conditions (e.g., pH 1), the stretching vibration of the imine bond (1610–1640 cm^−1^) was not distinguished due to the overlay by the amide I band. However, the stretching vibration of the C=O bond (aldehyde) from the hydrolyzed imine bonds for both as-spun and post-stretched DIF-72 fibers was detected at ~1740 cm^-1^, indicating the acid-induced hydrolysis of imine bonds within the DIF fibers, which resulted in the decreased mechanical performance. When the pH was recovered to 8, the stretching vibration of the C=O bond (aldehyde) decreased. This suggests that the imine bonds were reformed, thereby recovering the mechanical performance of DIF fibers to their initial state. Further control experiments showed that the ultimate tensile strength and toughness of degummed natural silkworm fibers were permanently decreased after being subjected to 2000 loading-unloading cycles or treated with a pH = 1 solution, which could not be recovered by treatment in a pH = 8 solution due to the irreversible destruction of internal structures within the silkworm fibers (Supplementary Figs. 31–32 and Supplementary Table 6). The distinct advantages in the recoverability of DIF fibers are attributed to the dynamic imine bonds within the DIF fibers. In a low pH solution, H^+^ facilitated the disassociation reaction of imine bonds, resulting in more fractured imine bonds and thus weakening the fiber strength. Therefore, the ultimate tensile strength gradually decreased as pH decreased. Under alkaline conditions, OH^—^ would facilitate the recondensation of amino and aldehyde groups, reconstructing the dynamic imine network within the fibers^8,50^, thereby recovering the fiber’s mechanics.

### Stability and reliability of the DIF fibers

The DIF fibers were stable for three and eight months under ambient conditions (25 °C, 30% RH, nonsterile) (Supplementary Fig. 33). Here the post-stretched DIF-72 fibers were investigated as an example. After storage for three months, the decreased mechanical strength was less than ~40 MPa. The toughness remained in the range of 63 to 74 MJ/m^3^. The slight decrease might be due to the plasticizing effect of water molecules entering the fiber’s interior upon exposure to air rather than the degradation. No distinguishable difference was observed in the mechanical performance of DIF-72 fibers after 3-, and 8-months’ storage, suggesting the good stability of DIF-72 fibers. Despite the slightly decreased ultimate tensile strength, the overall mechanical stability of DIF fibers is superior to other protein fibers under ambient conditions^51^. Such long-term mechanical stabilities of DIF-72 fibers might be related to the introduction of dynamic imine bonds. The rapid reaction between GA and lysine not only leads to the fast formation of uniformed DIF fibers, but also generates a cross-linking network that could protect fibers from degradation by environmental enzymes and bacteria, thus achieving long-term stability of the fibers.

**Fig. 4:**
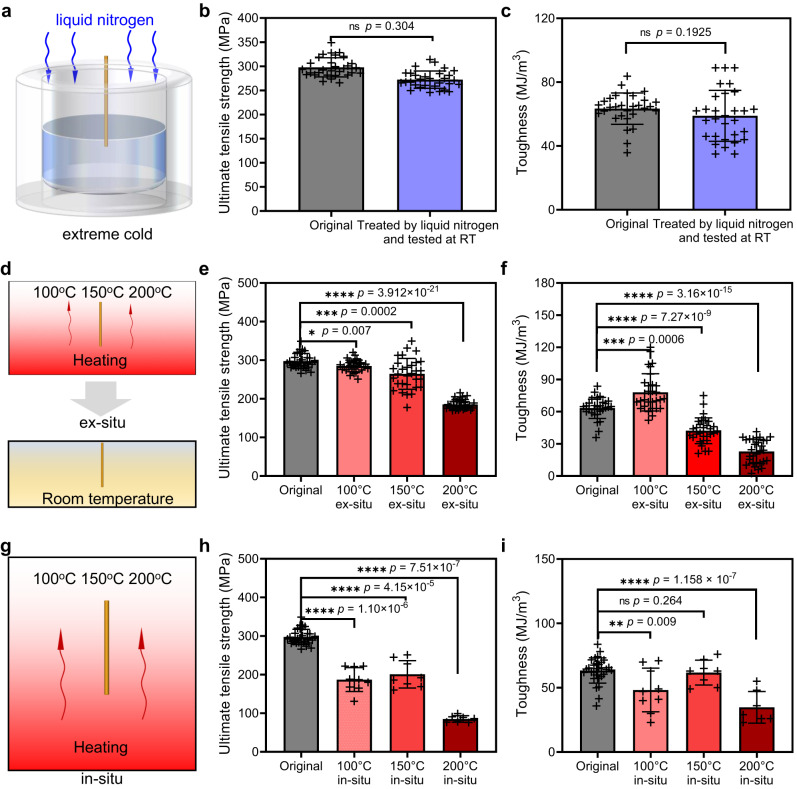
Stability and reliability of the DIF fibers under extreme conditions. **a** Schematics show the treatments of 100% post-stretched DIF-72 fibers under cold (cooling in liquid nitrogen at −196 °C for 12 h) conditions. **b**, **c** The ultimate tensile strength (**b**) and toughness (**c**) of post-stretched DIF-72 fibers at −196 °C for 12 h show no obvious decrease in mechanical performance after low-temperature treatment. ns no significant difference. All data (**b**, **c**) in the graphs are mean values of standard deviation (mean ± SD, *n* = 30). The result (**b**) is obtained by the statistical approach of *F*-tests. The result (**c**) is obtained by the statistical approach of two-way *T*-tests. **d** Schematics show the treatments of 100% post-stretched DIF-72 fibers under hot conditions (heating in an oven at 100, 150, and 200 °C for 12 h), and tested ex-situ at room temperature (RT). **e**, **f** The ultimate tensile strength (**e**) and toughness (**f**) of post-stretched DIF-72 fibers at ex-situ high temperatures (100, 150, and 200 °C) for 12 h. These values are only slightly decreased when heated at 100 and 150 °C in the system. When further increasing temperature to 200 °C, the mechanical performance of DIF-72 fibers was reduced significantly. All data (**e**, **f**) in the graphs are mean values of standard deviation (mean ± SD, *n* = 30). **g** Schematics showing the treatments of 100% post-stretched DIF-72 fibers by in situ high-temperature tests by DMA. **h**, **i** The ultimate tensile strength (**h**) and toughness (**i**) of post-stretched DIF-72 fibers at in situ high temperatures (100, 150, and 200 °C). The asterisks denote the statistical significance: *p < 0.05, ***p < 0.001, and ****p < 0.0001. All data (**h**, **i**) in the graphs are mean values of standard deviation (100 °C, mean ± SD, *n* = 9; 150 °C, mean ± SD, *n* = 8; 200 °C, mean ± SD, *n* = 7). Statistical analysis was performed with GraphPad Prism 8.0 and Origin 2021 software. The results (**b**, **c**), (**e**, **f**), (**h**, **i**) are obtained by the statistical approach of two-way *t*-tests.

Interestingly, the DIF-72 fibers demonstrated exceptional reliability even under extremely cold conditions, as illustrated in Fig. 4a. After being treated in liquid nitrogen for 12 h, the ultimate tensile strength and toughness of as-spun and post-stretched DIF-72 fibers showed no obvious decrease, suggesting their excellent stability at −196 °C (Fig. 4b, c, Supplementary Fig. 34, and Supplementary Table 7). We hypothesized that the introduction of imine-based cross-linking networks might prevent the approach or formation of ice clusters within the fibers at low temperatures, avoiding stress concentrations and cracking, thus maintaining the stability of the DIF-72 fibers^52,53^.

The mechanical performance of DIF-72 fibers at ex-situ high temperatures was further explored (Fig. 4d). In ex-situ high temperatures experiments, the as-spun/post-stretched DIF-72 fibers were repeatedly measured 30 times (Supplementary Figs. 35, 36, and Supplementary Table 7). Specifically, the DIF-72 fibers were treated at 100 °C, 150 °C, and 200 °C for 12 h and then tested ex-situ at RT. For the as-spun DIF-72 fibers, the ultimate tensile strength and toughness are 115.3 ± 9.0 MPa and 116.0 ± 15.7 MJ/m^3^ at 100 °C and gradually decreased to 98.9 ± 16.3 MPa and 99.0 ± 22.4 MJ/m^3^ (150 °C), which might be due to the high-temperature tolerance of K-protein system. In particular, tight entanglement of the random protein molecular chains and imine-induced networking in the as-spun DIF-72 fibers may confer thermal protection to the fibers, resulting in a slightly decreasing in ultimate tensile strength and toughness for the as-spun DIF-72 fibers. When further increasing temperature to 200 °C, these values decreased significantly to 83.4 ± 10.5 MPa and 17.9 ± 5.7 MJ/m^3^ due to the partly and irreversibly broken protein chain network. For the post-stretched DIF-72 fibers, at 100 °C, the ultimate tensile strength and toughness are 285.1 ± 14.8 MPa and 78.0 ± 17.1 MJ/m^3^, respectively. At 150 °C, these values are 264.5 ± 39.3 MPa and 42.2 ± 11.8 MJ/m^3^, which is still comparable to the original DIF-72 fibers at 25 °C. Such high-temperature resistance characteristics might be attributed to imine-based cross-linking networks within DIF fibers. These cross-linking networks might maintain the stability of the protein system to some extent in the range of 100 °C to 150 °C. In stark contrast, when degummed natural silkworm fibers were heated to 150 °C and then tested at RT, their ultimate tensile strength and toughness were irreversibly weakened (Supplementary Fig. 37), highlighting the thermal stability dominated by dynamic imine networking interactions within the DIF fibers. However, at 200 °C, the ultimate tensile strength and toughness significantly decrease to 185.2 ± 12.5 MPa and 22.9 ± 11.3 MJ/m^3^, respectively (Supplementary Figs. 35, 36). In this condition, the C=N (imine) bonds, C=O (aldehyde), and hydrogen bonds are broken, as observed in other system^54^. Besides, the stretching vibration of amide bonds changes obviously, especially for the amide III band, indicating that the protein network has been partly and irreversibly broken down at high temperatures (Supplementary Fig. 30), leading to an obvious decrease in fiber’s mechanical performance.

Next, in-situ high-temperature measurements of the mechanical performance of post-stretched DIF-72 fibers were performed by dynamic thermomechanical analysis (DMA) (Fig. 4d–f, Supplementary Fig. 38, and Supplementary Table 8). At 100 °C and 150 °C, the ultimate tensile strength of the post-stretched DIF-72 fibers gradually decreased to 187 ± 29 MPa and 200 ± 33 MPa, respectively (Fig. 4g, h, and Supplementary Fig. 38). Meanwhile, the toughness of these fibers was 48.2 ± 15.9 and 61.8 ± 9.0 MJ/m^3^, which were still comparable to the original post-stretched DIF-72 fibers. Such high-temperature resistance characteristics might be attributed to the high disassociation energy of dynamic imine bonds (147 kcal∙mol^−1^)^50^. These cross-linking networks might maintain the stability of the protein system to some extent in the range of 100 °C to 150 °C, resulting in in-situ high-temperature stability of the post-stretched DIF-72 fibers. In addition, even though some imine bonds may proceed to imine-imine metathesis or imine-amine exchange at high temperatures, they could still maintain the imine cross-linking density within the post-stretched DIF-72 fibers, thus exhibiting high-temperature mechanical stability. By contrast, the ultimate tensile strength and toughness of post-stretched DIF-72 fibers reduced significantly to 85.1 ± 7.9 MPa and 34.7 ± 11.4 MJ/m^3^ MPa when further increasing temperature to 200 °C. An increase in the damping factor was observed, suggesting that the protein chain networks are partly and irreversibly broken (Supplementary Figs. 39, 40). Overall, the DIF fibers exhibited excellent long-term stability and reliability under high/low temperatures.

Our observations show that the mechanical performance of DIF fibers in various experimental groups has different degrees of dispersion, but the overall dispersion is generally lower than the reported work^55^. Such lower viability might be due to the formation of dynamic imine bonds within DIF fibers. The protein utilized in our project has a randomly coiled structure with the lysine completely exposed. When protein solutions were mixed with GA (the molar ratio between lysine and GA is roughly approximately 1:4~6) through the wet-spinning technique, the rapid cross-linking reaction occurs between GA and lysine in protein, resulting in the production of homogeneous DIF fibers with smooth morphologies. Therefore, the mechanical properties of DIFs have low variabilities.

### Water-regulated actuation behaviors of the DIF fibers

**Fig. 5:**
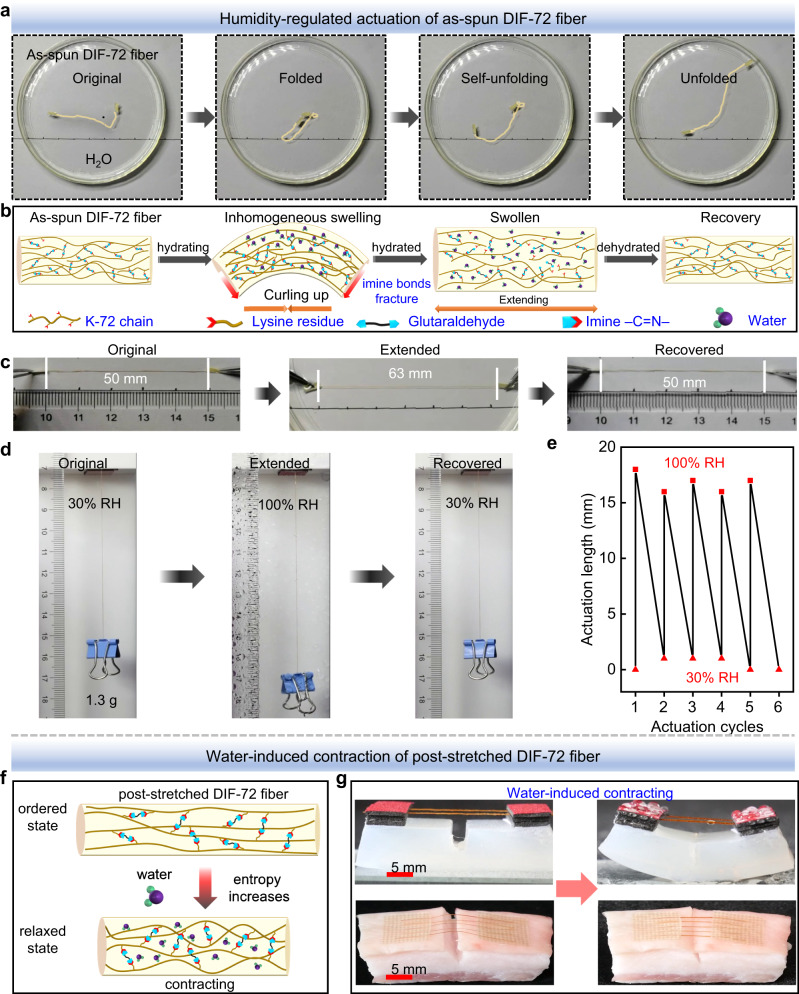
Water-regulated actuation behaviors of the as-spun DIF-72 fibers and the post-stretched DIF-72 fibers. **a** Self-folding and extending of the as-spun DIF-72 fiber in water (pH = 8) over time. A yellow line highlights the fiber. **b** Schematics illustrating the underlying mechanism for the self-folding and extending of the as-spun DIF-72 fiber in water over time. Self-folding was motivated by inhomogeneous swelling and hydrolysis of imine bonds, leading to a stress gradient in the fiber. When it was fully swollen, the fiber extended to relax the internal stress. **c** Length of a typical as-spun DIF-72 fiber before hydration, after hydration, and after dehydration. **d** Extension at high humidity and contraction at low humidity of the as-spun DIF-72 fiber. **e** Actuation cycles of reversible extension and contraction of the as-spun DIF-72 actuators triggered by humidity. **f** Water-induced contraction of the post-stretched fibers. The post-stretched DIF-72 fibers changed from an ordered state to a relaxed state upon hydration, leading to contraction. **g** Contraction of the post-stretched DIF-72 fiber bundles upon hydration sealed a 3 mm notch in agar gel (top) and porcine skin (bottom).

Amino acid residues in the amorphous regions play an important role in regulating the humidity response of protein fibers, such as twisting, curling, and contraction. The response process usually involves the destruction of original imine/hydrogen bonds and the formation of new hydrogen bonds owing to entering water molecules. In addition, the ordering of protein chains affects the interaction with water molecules and thus the behavior of fibers^56–58^. However, it was found that the as-spun DIF-72 fibers and post-stretched DIF-72 fibers exhibited different actuation behaviors when contacting water. For the as-spun DIF-72 fibers, protein chains were initially disordered. When in contact with an aqueous solution, self-folding and extending of as-spun DIF-72 fibers were observed: DIF fibers started to swell and curl quickly and eventually relieved to an extended state, as shown in Fig. 5a and Supplementary Movie 1. During hydration, water gradually diffuses into the protein fiber, leading to inhomogeneous swelling and thus curling the fiber^59^. Meanwhile, dynamic imine bonds were partially hydrolyzed and fractured by water, causing the elongation of the swollen fiber. Upon dehydration, the as-spun DIF-72 fibers recovered to their original state due to the reformed imine bonds (Fig. 5b). For a typical as-spun DIF-72 fiber of length = 50 mm, the fiber extended to 63 mm in water and recovered to its original length upon dehydration (Fig. 5c and Supplementary Movie 1). Unlike the self-folding and extending of as-spun DIF-72 fibers in water over time, the post-stretched DIF-72 fibers only exhibited rapid aggregation behavior when contacting water and contracted from 25 mm to 23 mm (Supplementary Fig. 41 and Supplementary Movie 2). This water-triggered contraction behavior is due to the relaxation of stretching-induced ordered protein chains to a less ordered state accompanied by an increase in entropy.

Based on the reversible extension and contraction of the as-spun DIF fibers upon hydration and dehydration, a humidity-responsive actuator was designed, which consisted of a swallow tail clip, an as-spun DIF-72 fiber with diameter = 150 μm and length = 83 mm, and a weight of 1.3 g. This actuator exhibited extraordinary reciprocating behaviors. It can reversibly lift and release the clip by 20% of its original length in many cycles when the fiber is dehydrated at 30% humidity and hydrated at 100% humidity, respectively (Fig. 5d, e and Supplementary Movie 3). While the post-stretched DIF-72 fibers can lift the clip by ~25% of its original length when the fiber is hydrated at 100% humidity, however, the fiber cannot release the clip back to its original place when it was dehydrated at 30% humidity (Supplementary Fig. 42 and Supplementary Movie 4). In the post-stretched DIF-72 fibers, protein chains were originally ordered. Therefore, unlike the as-spun DIF-72 fibers, the post-stretched DIF-72 fibers shrank when they were contacted with water due to the increased entropy^60,61^ (Fig. 5f). As observed, the twisted fiber bundles of 20 post-stretched fibers rapidly contracted when triggered by water and the contraction forces are strong enough to seal a 3 mm notch in agar gel or porcine skin (Fig. 5g and Supplementary Movies 5, 6). In contrast, the as-spun DIF-72 fiber bundles only extended to relax the internal stress upon hydration, failing to seal the notch in agar gel and porcine skin (Supplementary Fig. 43 and Supplementary Movies 7, 8). The absorption of water into highly-ordered and densely-packed protein chains disrupts original interchain imine bonds and hydrogen bonds, and new hydrogen bonds are formed between water and hydrophilic groups (-C=O, -C-NH-, -NH_2_) in the protein chains, causing the entropy-driven curling and contraction of protein chains^62^, thereby leading to contraction at high humidity and no extension at low humidity. In addition, when the post-stretched DIF-72 fibers were weaved into a fiber mesh, a weight = 3 g metal ball falling from a height of 25 cm was rebounded essentially to its original height, suggesting the excellent impact resistance of the post-stretched DIF-72 fibers (Supplementary Fig. 44 and Supplementary Movie 9).

## Discussion

By combing protein engineering and dynamic imine bonds, we presented DIFC as an alternative strategy for the rational design of robust and tough protein fibers. In contrast to conventional protein fibers with inferior environmental adaptability, the reversible disassembly and reassembly properties of imine bonds within the protein fibers allowed DIF fibers with tunable mechanical performances under different conditions. Especially, the DIF fibers exhibited excellent mechanical stability and recoverability against extreme environments. The imine bonds with a high disassociation energy can resist extreme conditions and maintain a high interacting density within the fibers, thus contributing to robust mechanical stability. In particular, owing to the hydration inhomogeneity and dynamic imide chemistry within the as-spun DIF fibers, their versatile self-motivated actuation modes were achieved. Furthermore, water-induced contraction of the post-stretched DIF fibers can be utilized to seal the notch in porcine skin or agar gel. The DIF fibers’ mechanical performance and low cytotoxicity enable their potential for biomedical applications. However, we do need to further optimize the system to enable practical biomedical translation. For example, we might avoid glutaraldehyde crosslinking agents by using alternatives, such as in-situ chitosan ring-opening crosslinking. In addition, other fiber spinning techniques in the absence of aldehyde reaction can be developed. Overall, our strategy enables the precise engineering of intra- and inter-molecular interactions via the manipulation of amino acid sequences and the introduction of dynamic amine bonds between protein chains, thus achieving high mechanical performances and diverse functionalities and intriguing new inspirations for the design of protein fibers for high-tech applications.

## Methods

### Materials

All biochemicals for cloning and K-proteins expression, such as LB medium, salts, antibiotics as well as inducer compounds, were used as received (from Sigma-Aldrich) without any further purification. Glutaraldehyde (GA) were purchased from Aladdin Co., Ltd. (China). Other chemicals were obtained from Sigma-Aldrich (China). The water used in this research (18.2 MΩ cm at 25 °C) was from a Milli-Q ultrapure water system (Merck, Germany). DNA marker, 10× loading buffer were purchased from TaKaRa Biotechnology. Alpha-cyano-4-hydroxycinnamic acid and sinapinic acid was used as matrix during MALDI mass spectrometry and was purchased from Thermo Scientific (Waltham, MA).

### Expression of recombinant K-proteins

The complete sequences and molecular weights of K-36, K-72, K-144, and K-144cys proteins are shown in Supplementary Table 1. The plasmids containing the DNA sequences of the K-proteins were prepared according to literature^19^ and were confirmed by DNA sequencing. The pET25b expression vectors containing the K-protein genes were transformed into *E. coli* BLR (DE3) cells (Novagen). The protocols for gene expression and protein production refer to the literature^19^.

### Scale-up fermentation of K-72 protein

The mass fermentation of K-72 protein was optimized in a 100 L fermentor. First, *E. coli* BLR21-K-72 cells were transferred into a sterilized LB culture medium (10 g/L peptone, 5 g∙L^−1^ yeast extract, 10 g/L sodium chloride, and 100 μg∙mL^−1^ ampicillin) and incubated in a shaker at 37 °C under 220 rpm∙min^−1^ shaking for 8 h to obtain fermentation seed. Next, the seed was inoculated in the fermentation medium in the tank (Supplementary Table 2) at an inoculation ratio of 1.67% (seed: medium = 1 L: 60 L) and an initial optical density (OD) of 0.3~0.5. After adding ampicillin with a final concentration of 200 μg/mL, cell enrichment was started with a temperature of 37 °C, stirring rate of 500 rpm∙min^−1^, air supply of 30 L/min, tank pressure of 0.04 MPa and pH of 6.9. The pH was kept at around 6.9 by the automatic addition of aqueous ammonia. After 5 h of fermentation, glucose was supplied by a peristaltic pump at a stable rate to control dissolved oxygen >20%. When the OD reached ~30, isopropyl-beta-D-thiogalactopyranoside (IPTG) was added into the fermentation medium with a final concentration of 0.5 mmol∙L^−1^ to induce protein expression. The glucose supply rate was maintained until 2 h before the end of fermentation. After fermentation, the fermentation broth was centrifuged in a high-speed centrifuge to acquire *E. coli* cells, which expressed K-72 protein.

### Purification of recombinant K-proteins

First, collected *E. coli* cells were suspended in a lysing solution (50 mm sodium phosphate buffer of pH = 7.4, 500 mm NaCl, 20 mm imidazole, 1 mg/mL lysozyme, 5 μg/mL DNase, and 30 mm MgCl_2_) to an OD of 100 and then broken by a cell disrupter (Constant Systems Limited, Daventry, UK). Cell debris was removed by centrifugation. Proteins were purified from the supernatant under ambient conditions by Ni Sepharose 6XFF column (General Electric Company). Protein-containing fractions were collected by linear gradient elution with washing buffer (50 mm sodium phosphate buffer of pH = 7.4, 500 mm NaCl, and 20 mm imidazole) and elution buffer (50 mm sodium phosphate buffer of pH = 7.4, 500 mm NaCl, and 350 mm imidazole), and then dialyzed in a solution with 50 mm sodium phosphate buffer of pH = 7.4 and 50 mm NaCl for 2 h. After dialysis, the protein-containing fractions were purified by an SP HP column (General Electric Company) and collected by linear gradient elution with washing buffer (50 mm sodium phosphate buffer of pH = 7.4 and 50 mm NaCl) and elution buffer (50 mm sodium phosphate buffer of pH = 7.4 and 2 m NaCl). The obtained protein-containing fractions were further purified by a desalting column (General Electric Company) and collected using water. Purified proteins were frozen in liquid nitrogen, lyophilized, and stored at −80 °C before use. The purity and molecular weight of proteins were confirmed by sodium dodecyl sulfate-polyacrylamide gel electrophoresis (SDS-PAGE) in a 12% polyacrylamide gel using a 6x protein loading buffer (Beijing TransGen Biotech Corporation Limited).

### Mass spectrometric analysis of K-proteins

All K-protein samples were dissolved in ultrapure water at a concentration of 1 mg/mL for mass spectrometric analysis (autoflexIII MALDI-TOF/TOF, Bruker, German).

### Proton nuclear magnetic resonance analysis of K-proteins

All K-protein samples were dissolved in D_2_O at a concentration of 16 mg/mL for ^1^H NMR analysis (Bruker, 500 MHz).

### High-performance liquid chromatography-mass spectrometric analysis of K-proteins

All K-proteins were dissolved in ultrapure water at a concentration of 0.5 mg/mL for HPLC-MS analysis (ZORBAX RRHD Eclipse Plus C8, 95 Å, 2.1 × 100 mm, 1.8 µm, positive mode). Water/acetonitrile/0.1% formic acid was employed as an elution buffer.

### High-performance liquid chromatography analysis and final purification yield determination of K-proteins

All K-proteins were dissolved in ultrapure water at a concentration of 2.0 mg/mL for HPLC analysis (Agilent Bio SEC-3, 150 Å, 4.6 × 50 mm, 3 µm, positive mode). 150 mm Na_3_PO_4_ (pH 7.4) was employed as an elution buffer. Moreover, combined with the purity determined by HPLC, we can calculate the final purification yield of K protein by weighting the lyophilized protein. For example, 100 L cultures of K-72 protein were processed by chromatographic purification, after weighting the lyophilized protein the final yield of K-72 protein was determined.

### Cytotoxicity of K-proteins

In brief, 293 T cells were seeded in a 96-well plate at a density of 10^4^ cells per well and incubated overnight at 37 °C and 5% CO_2_. The culture medium was removed and replaced with different concentrations of K-72 protein. After being incubated for 24 h, 10 µL Cell Counting Kit-8 (APEXBIO) was added to each well following the manufacturer’s protocol. The absorbance at 450 nm was recorded after incubation for 1 h, and the relative cell activity was assessed. Statistical analysis was performed with GraphPad Prism 8.0.

### Circular dichroism analysis of K-proteins

The conformations of K-proteins were characterized by CD using a J-820 spectropolarimeter. Prior to each set of CD measurements, the concentrated proteins were diluted to 0.2 mg/mL. H_2_O was used as a calibrant for the CD measurements. To ensure reproducibility, measurements were conducted in triplicate across wavelengths ranging from 190 to 260 nm, using a 100 nm/min scanning rate and 1 nm bandwidth.

### Preparation of dynamic imine fibers (DIF fibers)

Designed K-proteins do not contain any terminal domains, which are essential for protein aggregation. K-proteins possess repeat sequences of VPGVG(VPGKG)_9_ and thus many highly reactive amino groups of lysine residues. Therefore, amino groups will condensate with glutaraldehyde in the coagulation bath and a dynamic imine network is formed, leading to the formation of integrated K-protein fibers. All lyophilized K-proteins were dissolved in deionized water at the same concentration of 200 mg/mL. When dissolved, K-proteins tend to be in a disordered state. The protein solutions were extruded from the needle with an inner diameter = 210 μm and an outer diameter = 420 μm into an aqueous solution with 1% (w/w) glutaraldehyde (J&K Scientific Limited, China) using a syringe pump (LSP01-3A, Longer, China). The low GA concentration (1%) ensures the monomeric form of GA molecules^63^, which are highly reactive to form imine bonds between K-proteins during the spinning process. The protein extrusion rate could range from 5 μL/min to 30 μL/min. If not specified, a typical extrusion rate of 15 μL/min was used. As-spun fibers were automatically collected on a rotor driven by a motor (86HBP115AL4S, Beijing Haijiejiachuang Technology Co., Ltd, China) and dried on a rotating collector after the proteins in the solution were cross-linked by glutaraldehyde in the coagulation bath. The rotor collection speed could vary from 1.5 m·min^−1^ to 6.4 m·min^−1^. If not specified, a typical collection speed of 6.4 m/min was used. Before characterization, collected fibers were dried under ambient conditions for 3 h, which facilitated the complete cross-linking of GA in the fibers and the evaporation of residual GA. Post-stretched fibers were prepared by hydrating as-spun fibers in water and then drawing the fibers by 100% using a tensile tester at 10 mm/min (FAVIMAT+ instrument, Textechno, Germany).

### Scanning electron microscopy (SEM)

Surface morphology and cross-section of DIF fibers were observed by scanning electron microscopy (HITACHI S-4800) operated at 10 kV. Samples were mounted on specimen stubs using conductive double-sided adhesive tapes and sputtered with gold for 30 s.

### Polarized optical microscopy (POM)

The molecular order and birefringence of DIF fibers were investigated by a polarized optical microscope (Nikon, ECLIPSE LV100N POL).

### Small-angle X-ray scattering (SAXS)

Small-angle x-ray scattering (SAXS) of DIF fibers was carried out using synchrotron radiation at BL19U2 station of Shanghai Synchrotron Radiation Facility (SSRF). The x-ray wavelength was 0.889 nm with an energy of 13.95 keV. The sample-to-detector distance was 2753 mm. Individual DIF was mounted on a hollow specimen holder along the vertical direction. The exposure time for each measurement was 5 s. SAXS analysis was carried out by Fit 2D.

### Tensile tests of DIF fibers

All DIF fibers were air-dried for 3 h before testing. Tensile tests of different fibers were acquired using a tensile tester (FAVIMAT+ instrument, Textechno, German). The diameter of fibers was measured using POM before the tensile test. The cross sections of fibers are essentially circular. The fibers were fixed between two miniclamps along the vertical direction with the aid of pneumatic flow. The sample length is 5 mm. The stretching speed is 10 mm/min. Engineering strain (${{{{{\rm{strain}}}}}}=\frac{\Delta l}{{l}_{0}}$) and engineering stress (${{{{{\rm{stress}}}}}}=\frac{F}{\pi {{r}_{0}}^{2}}$) are used, where $\Delta l$ is the displacement length, ${l}_{0}$ is the original fiber length, F is the tensile force, and ${r}_{0}$ is the original fiber radius. The stress-strain curves were not averaged and smoothened. Young’s modulus was determined as the slope of the stress-strain curve within the linear elastic deformation range at 1% strain. Toughness was calculated by integrating the area under the stress-strain curve using Origin 2021. If not specified, 30 samples were measured for each group. The loading-unloading cycles of post-stretched fibers were performed at 5% and 20% strain at room temperature. Commercial degummed silkworm fibers (*Bombyx mori Linnaeus*, Taobao, China) were purchased and tested without any further treatments. The testing parameters are the same as those used for DIF samples.

### Thermogravimetric analysis (TGA)

TGA was performed to test the thermostability of DIF fibers using a thermal analyzer (STA 449F3) under an N_2_ flow of 50 mL/min at a heating rate of 10 °C∙min^−1^ from 30 to 500 °C.

### Dynamic thermomechanical analysis (DMA)

DMA 850 was used for the in-situ tests of the tensile behaviors of DIF fibers at 100 °C, 150 °C, and 200 °C. The curves of storage modulus, loss modulus, and damping factor = loss modulus/storage modulus (tan δ) versus temperature were obtained by DMA.

### Fourier transform infrared analysis (FTIR)

FTIR spectroscopy of the samples was collected using a Germany Bruker INVENIO-R FT-IR spectrometer. To gain an acceptable signal-to-noise ratio, 128 scans with a resolution of 4 cm^−1^ have been accumulated. ATR spectrum uses diamond reflection crystal.

### Cytotoxicity of the DIF fibers

Diverse cell lines were employed to evaluate the cytotoxicity of the DIF fibers (DIF-36, DIF-72, DIF-144, and DIF-144cys). Human embryonic kidney cells 293 T (catalog number: CL-0005, purchased from Procell Life Science & Technology, China) and Mouse hippocampal neuronal cells HT22 (catalog number: CL-0697, purchased from Procell Life Science & Technology, China) were cultured in DMEM medium supplemented with 10% FBS, while Mouse fibroblast cells L929 (catalog number: CL-0137, purchased from Procell Life Science & Technology, China) were cultured in 1640 medium supplemented with 10% FBS. All cells were seeded at a density of 10^5^ cells per well in a 12-well plate and co-cultured with UV-sterilized fibers for 24 h. 1 µL of calcein-AM staining and 1 µL of propidium iodide (PI) were added to each well and incubated for 20 min. Subsequently, dyes were removed and washed with PBS buffer, and the images of cells with DIFs were photographed by LSCM at an emission wavelength of 488 nm for AM and 561 nm for PI. The cell lines used in this article are not listed in misidentified cell lines maintained by the International Cell Line Authentication Committee.

### Reporting summary

Further information on research design is available in the Nature Portfolio Reporting Summary linked to this article.

### Supplementary information

Supplementary Information

Reporting Summary

Description of Additional Supplementary Files

Supplementary Movie 1

Supplementary Movie 2

Supplementary Movie 3

Supplementary Movie 4

Supplementary Movie 5

Supplementary Movie 6

Supplementary Movie 7

Supplementary Movie 8

Supplementary Movie 9

### Source data

Source Data

## Data Availability

The authors confirm that the data supporting the findings of this study are available within the article (Figs. 1–5) and/or its supplementary materials (Supplementary Figs. 1–44, Tables 1–8, and Movies 1–9). Source data are provided in this paper. The tensile test, CD analysis, HPLC, DMA, TGA, SDS-PAGE, HPLC-TOF MS, cytotoxicity, SAXS data used in this study are provided in the Source Data file. Source data are provided with this paper.
