# Supplementary material for: Protein fibers with self-recoverable mechanical properties via dynamic imine chemistry
Source: Nat Commun. 2023 Sep 2;14:5348. doi: 10.1038/s41467-023-41084-1 (PMC10475138; doi:10.1038/s41467-023-41084-1)
Supplement: Supplementary file 1 — Supplementary Information [file 41467_2023_41084_MOESM1_ESM.pdf]

Supplementary Information

Protein fibers with self-recoverable mechanical properties via dynamic imine chemistry

Jing Sun[1#], Haonan He[2,3#], Kelu Zhao[2,3#], Wenhao Cheng[2,3], Yuanxin Li[2,3], Peng Zhang[2,3], Sikang Wan[2], Yawei Liu[3], Mengyao Wang[2,3], Ming Li[2,3], Zheng Wei[2,3], Bo Li[2], Yi Zhang[2,3], Cong Li[4], Yao Sun[2], Jianlei Shen[4], Jingjing Li[3], Fan Wang[3], Chao Ma[2], Yang Tian[1], Juanjuan Su[5]*, Dong Chen[6]*, Chunhai Fan[4], Hongjie Zhang[2,3], Kai Liu[2,3]*

[1]School of Chemistry and Molecular Engineering, Shanghai Engineering Research Center of Molecular Therapeutics and New Drug Development, East China Normal University, Shanghai, 200241, China.

[2]Engineering Research Center of Advanced Rare Earth Materials (Ministry of Education), Department of Chemistry, Tsinghua University, 100084 Beijing, China. Email: kailiu@tsinghua.edu.cn.

[3]State Key Laboratory of Rare Earth Resource Utilization, Changchun Institute of Applied Chemistry, Chinese Academy of Sciences, 130022 Changchun, China.

[4]Frontiers Science Center for Transformative Molecules, School of Chemistry and Chemical Engineering, and Institute of Molecular Medicine, Renji Hospital, School of Medicine, Shanghai Jiao Tong University, Shanghai, 200240, China.

[5]Center of Materials Science and Optoelectronics Engineering, College of Materials Science and Optoelectronic Technology, University of Chinese Academy of Sciences, Beijing 100049, China; Email: sujuanjuan@ucas.ac.cn.

[6]College of Energy Engineering, Zhejiang University, Hangzhou 310027, China. Email: chen_dong@zju.edu.cn.

[#]These authors contributed equally: Jing Sun, Haonan He, and Kelu Zhao

Supplementary Tables and Figures

Supplementary Table 1 General information of K-proteins used in this work. M^* denotes molar mass calculated by ProtParam (https://web.expasy.org/protparam/). $M^\#$ denotes molar mass determined by mass spectroscopy in **Supplementary Figure 1**.

Protein	Sequence	M^* (Da)	$M^\#$ (Da)
K-36	$MGAGP[(GVGVP)(GKGVP)_9]_4GWPH_6$	18888	18891
K-72	$MGAGP[(GVGVP)(GKGVP)_9]_8GWPH_6$	36444	36281
K-144	$MGAGP[(GVGVP)(GKGVP)_9]_{16}GWPH_6$	71294	70973
K-144cys	$MCGAGP[(GVGVP)(GKGVP)_9]_{16}GWPH_6C$	71860	72019

Supplementary Table 2 Optimized formula for the mass fermentation of K-72 proteins in 100 L fermentor.

Material	Concentration
KH_2PO_4	6.67 g/L
Peptone	13 g/L
Citric Acid·H_2O	3.25 g/L
$CaCl_2$·H_2O	0.05 g/L
$MgSO_4$·$7H_2O$	2.5 g/L
$FeSO_4$·$7H_2O$	5 mg/L
$ZnSO_4$·$7H_2O$	3.8 mg/L
$MnSO_4$·H_2O	0.33 mg/L
$CuSO_4$·$5H_2O$	0.1 mg/L
$CoCl_2$·$6H_2O$	0.1 mg/L
Defoamer	1 mL/L
Aqueous Ammonia	28%
Ampicillin	1 mL/L
Isopropyl-beta-D-thiogalactopyranoside (IPTG)	0.5 mM
Glucose	650 g/L

Supplementary Table 3 Summary of mechanical properties of DIF fibers.

DIF		Ultimate tensile strength (MPa)	Modulus (GPa)	Toughness (MJ/m^3)	Tensile strain (%)	Diameter (μm)
DIF-36	As-spun	98.9 ± 3.3	2.5 ± 0.4	76.0 ± 9.3	105.1 ± 12.7	24.9 ± 1.5
	Post-stretched	210.8 ± 14.4	2.6 ± 0.5	39.8 ± 8.5	27.8 ± 5.1	19.4 ± 2.8
DIF-72	As-spun	103.8 ± 9.1	2.3 ± 0.3	100.8 ± 15.6	165.7 ± 26	23.8 ± 2.5
	Post-stretched	297.7 ± 19.8	3.5 ± 0.6	63.4 ± 9.8	41.6 ± 9.5	16.6 ± 0.9
DIF-144	As-spun	130.4 ± 5.6	3.6 ± 0.4	140.8 ± 15.7	142.8 ± 14.5	21.8 ± 1.6
	Post-stretched	367.1 ± 12.2	4.4 ± 0.6	73.9 ± 17.0	30.0 ± 5.7	19.6 ± 1.3
DIF-144cys	As-spun	144.1 ± 16.3	2.9 ± 0.6	143.8 ± 24.5	141.9 ± 18.2	24.4 ± 4.2
	Post-stretched	396.3 ± 18.7	4.9 ± 0.6	76.2 ± 18.1	28.1 ± 5.5	22.8 ± 2.6

Supplementary Table 4 Summary of mechanical properties of fatigued and recovered DIF-72 fibers.

DIF-72	Ultimate tensile strength (MPa)	Modulus (GPa)	Toughness (MJ/m^3)	Strain (%)	Diameter (μm)
Fatigued	253.6 ± 13.5	3.5 ± 0.6	39.7 ± 8.3	24.1 ± 5.1	18.1 ± 0.8
Recovered	288.2 ± 5.5	4.0 ± 0.7	65.4 ± 11.0	33.2 ± 5.2	16.9 ± 1.0

Supplementary Table 5 Summary of pH-dependent performances of the DIF-72 fibers.

DIF-72		Ultimate tensile strength (MPa)	Modulus (GPa)	Toughness (MJ/m^3)	Tensile strain (%)	Diameter (μm)
pH=7 treated	As-spun	106.7 ± 19.5	3.3 ± 3.1	90.5 ± 20.1	128.6 ± 21.8	26.1 ± 3.1
	Post-stretched	277.2 ± 29.2	2.7 ± 0.5	64.2 ± 14.7	36.5 ± 7.6	17.9 ± 1.5
pH=7→8	As-spun	126.3 ± 7.7	2.7 ± 0.5	122.8 ± 15.9	134.9 ± 17.4	24.9 ± 2.4
	Post-stretched	285.8 ± 17.9	3.3 ± 0.5	63.2 ± 15.4	32.7 ± 6.6	17.3 ± 1.5
pH=5 treated	As-spun	100.9 ± 19.3	2.1 ± 0.2	90.1 ± 17.9	146.8 ± 25.6	25.8 ± 2.8
	Post-stretched	220.2 ± 33.3	2.5 ± 0.4	48.5 ± 15.5	35.7 ± 7.1	18.4 ± 1.8
pH=5→8	As-spun	119.9 ± 20.9	2.0 ± 0.3	116.0 ± 29.9	152.1 ± 27.4	24.3 ± 1.2
	Post-stretched	299.8 ± 11.4	3.6 ± 1.6	85.6 ± 15.2	44.2 ± 7.3	18.7 ± 0.8
pH=3 treated	As-spun	91.4 ± 14.1	2.2 ± 0.2	111.2 ± 22.6	151.3 ± 30.1	24.8 ± 2.5
	Post-stretched	213.3 ± 25.6	2.7 ± 0.3	52.3 ± 10.1	31.9 ± 5.4	18.5 ± 1.3
pH=3→8	As-spun	118.6 ± 14.1	2.4 ± 0.5	97.0 ± 16.3	124.2 ± 19.3	26.1 ± 5.2
	Post-stretched	300.0 ± 21.5	2.9 ± 0.7	74.5 ± 17.7	40 ± 7.8	16.6 ± 1.0
pH=1 treated	As-spun	88.8 ± 13.9	1.9 ± 1.3	98.8 ± 18.2	173.3 ± 23.8	24.4 ± 2.8
	Post-stretched	176.7 ± 19.1	2.2 ± 0.4	34 ± 7.2	31.4 ± 4.8	18.4 ± 0.9
pH=1→8	As-spun	117.1 ± 15.4	2.5 ± 0.3	97.6 ± 24.5	119.5 ± 27.9	25.4 ± 2.3
	Post-stretched	288.7 ± 36.7	2.8 ± 0.4	69.4 ± 16.5	38.3 ± 6.4	17.2 ± 0.9

Supplementary Table 6 Summary of mechanical performances of degummed natural silkworm silks under different treatments.

Degummed natural silkworm silk	Ultimate tensile strength (MPa)	Toughness (MJ/m^3)
Original	664 ± 42	47 ± 13
Fatigued	393 ± 44	30 ± 4
Treated by pH=8 solution after fatigued	438 ± 57	27 ± 9
Treated by pH=1 solution	389 ± 34	18 ± 5
Treated by pH=8 solution after treated by pH=1 solution	417 ± 31	30 ± 8
150°C treated	429 ± 99	22 ± 9

Supplementary Table 7 Summary of ex-situ tests of the DIF-72 fibers at high/low temperatures.

DIF-72		Ultimate tensile strength (MPa)	Modulus (GPa)	Toughness (MJ/m^3)	Tensile strain (%)	Diameter (μm)
100°C	As-spun	115.3 ± 9.0	2.2 ± 0.4	116.0 ± 15.7	160.9 ± 15.7	25.8 ± 1.3
	Post-stretched	285.1 ± 14.8	3.1 ± 0.4	78.0 ± 17.1	43.2 ± 6.0	18.2 ± 1.0
150°C	As-spun	98.9 ± 16.3	2.7 ± 0.4	99.0 ± 22.4	119.3 ± 26.8	26.7 ± 1.9
	Post-stretched	264.5 ± 39.3	3.4 ± 0.5	42.2 ± 11.8	26.9 ± 5.2	18.7 ± 0.9
200°C	As-spun	83.4 ± 10.5	1.0 ± 0.7	17.9 ± 5.7	25.6 ± 6.2	23.5 ± 3.1
	Post-stretched	185.2 ± 12.5	4.3 ± 1.0	22.9 ± 11.3	15.7 ± 5.9	15.9 ± 0.5
-196°C	As-spun	107.3 ± 11.1	2.9 ± 0.3	103.2 ± 16.3	128.5 ± 13.9	24.0 ± 1.8
	Post-stretched	272.4 ± 17.7	3.2 ± 0.4	58.9 ± 15.7	34.6 ± 7.3	18.0 ± 1.4

Supplementary Table 8 Summary of in-situ tests of the post-stretched DIF-72 fibers at high temperatures.

DIF-72	Ultimate tensile strength (MPa)	Modulus (GPa)	Toughness (MJ/m^3)	Tensile strain (%)	Diameter (μm)
100°C	187.1 ± 29.1	2.2 ± 0.4	48.2 ± 15.9	23.0 ± 6.2	18.7 ± 1.2
150°C	200.9 ± 33.2	2.9 ± 1.1	61.8 ± 9.0	25.6 ± 6.0	19.7 ± 1.2
200°C	85.1 ± 7.9	1.2 ± 0.3	34.7 ± 11.4	29.2 ± 7.6	20.4 ± 1.2

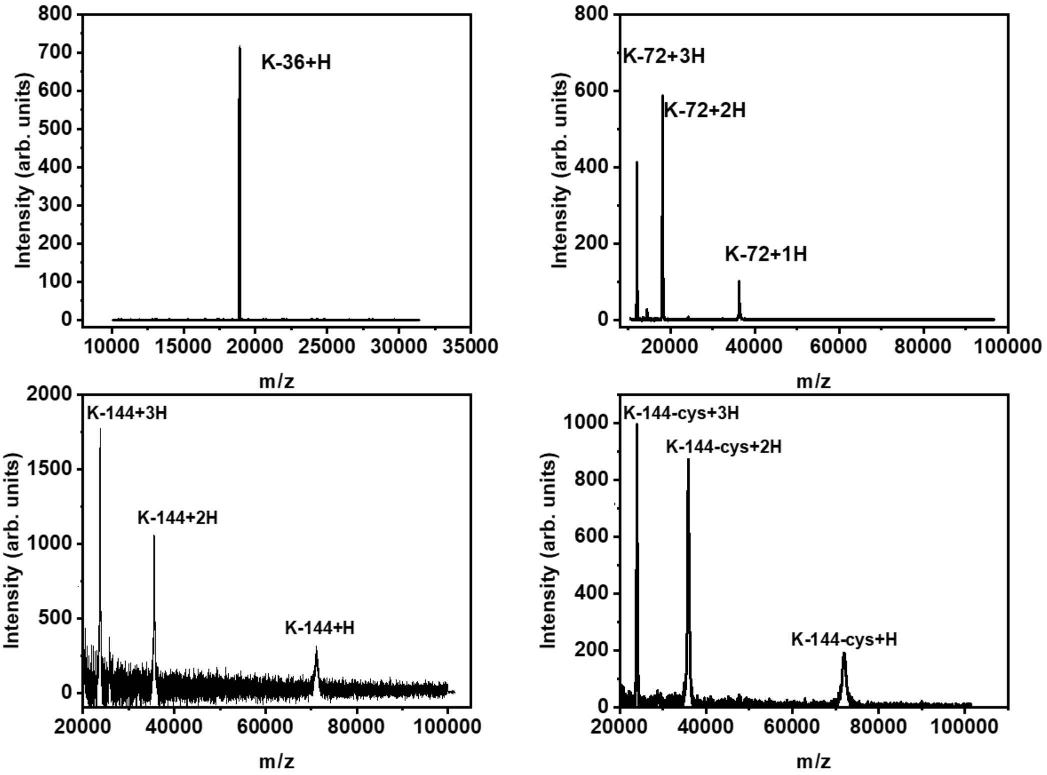

Supplementary Figure 1 Matrix-assisted laser desorption/ionization time-of-flight (MALDI-TOF) mass spectra of the K-protein samples.

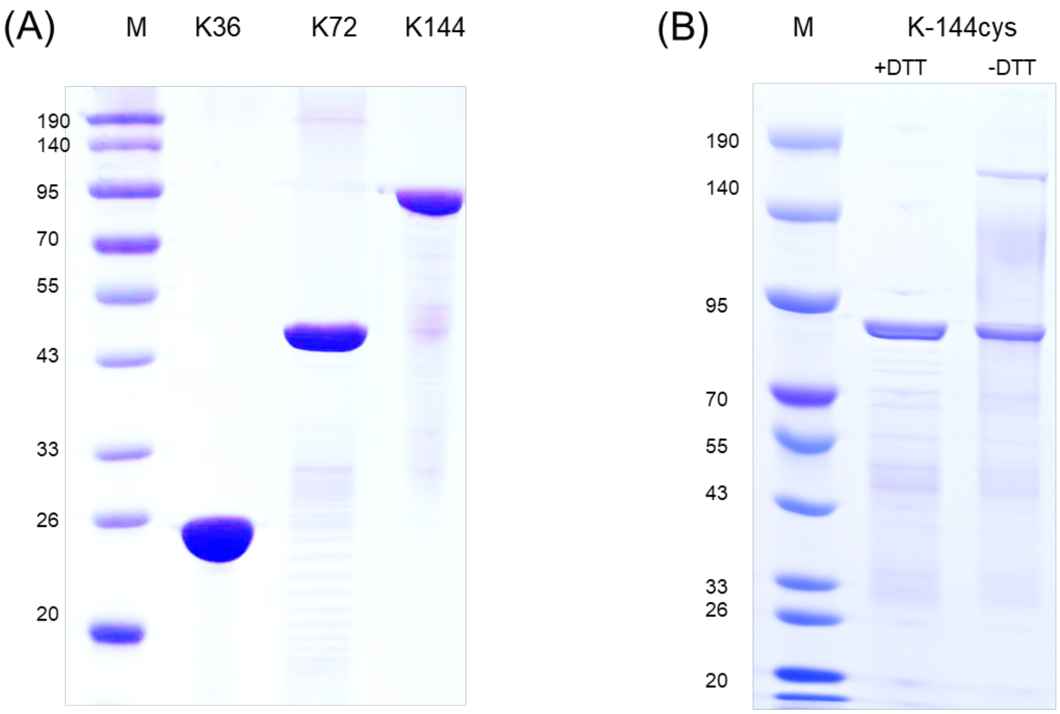

Supplementary Figure 2 (**A**) SDS-PAGE analysis of purified K-36, K-72, and K-144 proteins by Coomassie staining. (**B**) SDS-PAGE analysis of K-144cys proteins with and without dithiothreitol (DTT) by Coomassie staining. A dimerization band is observed in the gel of K-144cys proteins when loading the buffer without DTT.

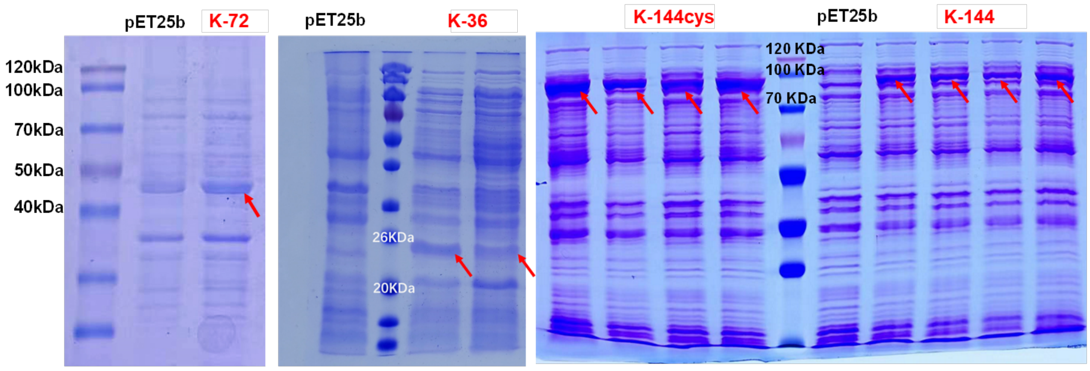

Supplementary Figure 3 Sodium dodecyl sulfate-polyacrylamide gel electrophoresis (SDS-PAGE) analysis of pET25b (protein pattern of *E. coli* without the expression plasmid) and unpurified K proteins. The red arrows represent the target K proteins.

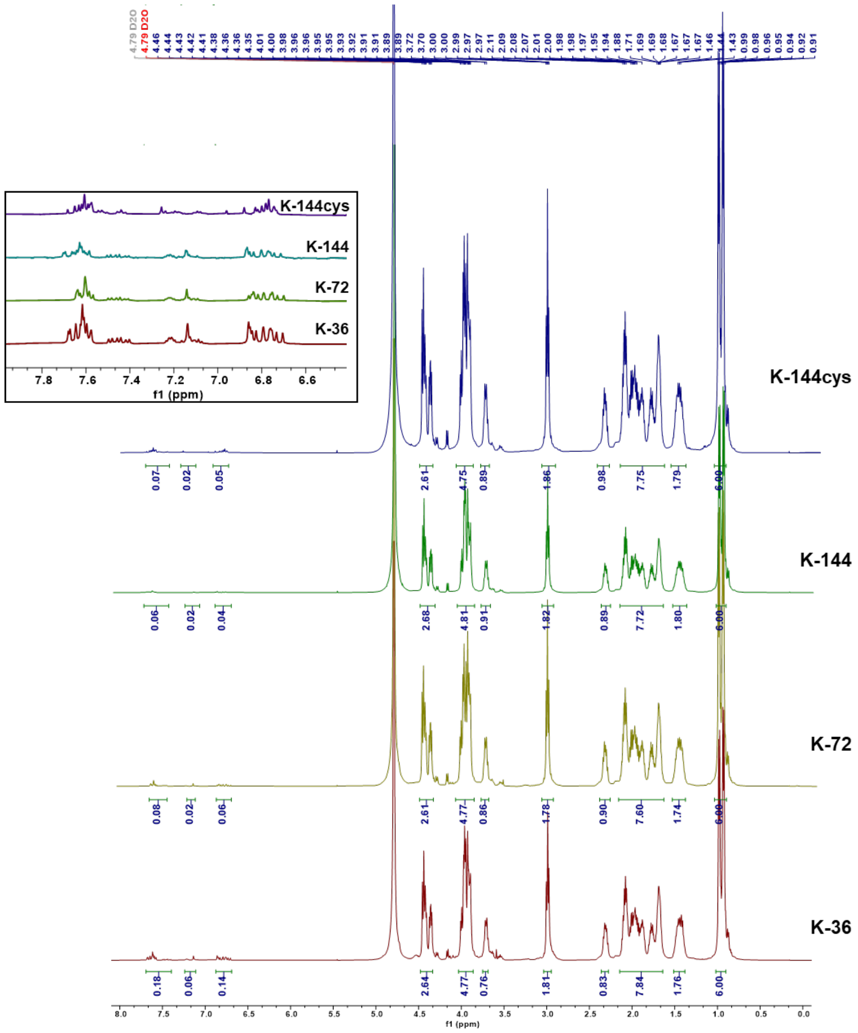

Supplementary Figure 4 ^{1}H NMR (500 MHz, D_2O) characterization of different recombinant proteins (16 mg·mL^{-1}), from bottom to top: K-36, K-72, K-144, and K-144cys. There is no distinguishable difference between the spectra of K-36, K-72, K-144, and K-144cys proteins, since the recombinant proteins contain the same repeat unit of VPGVG(VPGKG)$_9$ and a tryptophan (W) at the end of amino acid sequence. The inset is magnified proton signals in the chemical shift between 6.5 ppm to 7.5 ppm, which belong to the aromatic proton signal of tryptophan. The tryptophan residues can be found in all K-proteins, which is the reason for the detection of signals between 6.5 and 7.5 ppm in all K-proteins.

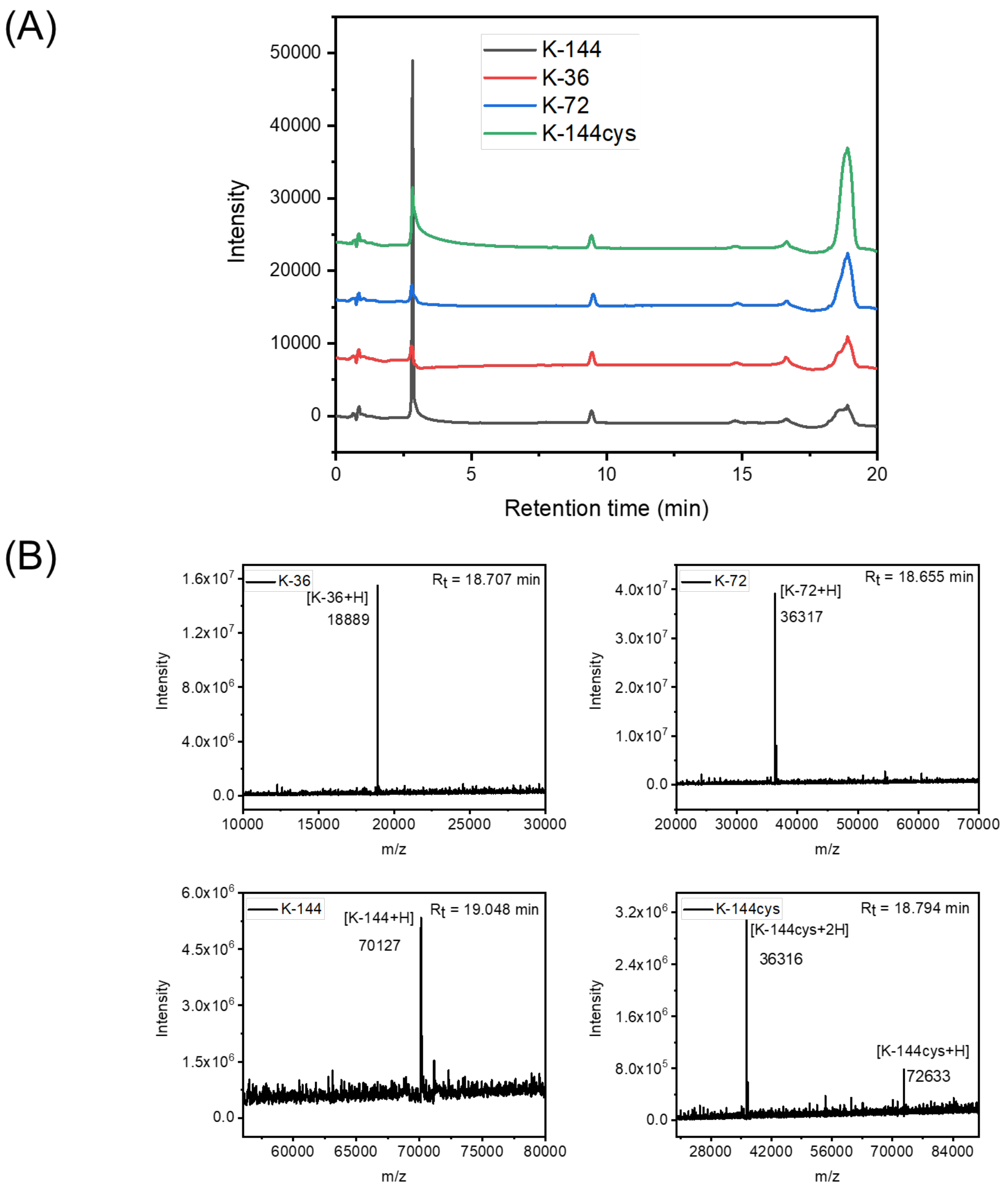

Supplementary Figure 5 HPLC-MS analysis of K-proteins tested at 0.5 mg·mL^{-1}. (**A**) The HPLC chromatograph of K-36, K-72, K-144, and K-144cys proteins was eluted at around 18.7 min, 18.6 min, 19.0 min, and 18.8 min, respectively. The peaks at the retention time of 2.8 min and 9.4 min were impurities from the elution buffer. (**B**) The mass results of K-proteins show a molecular weight of 18889 Da, 36317 Da, 70127 Da, and 72633 Da for K-36, K-72, K-144, and K-144cys proteins, respectively, which is in good agreement with the results of MALDI-TOF.

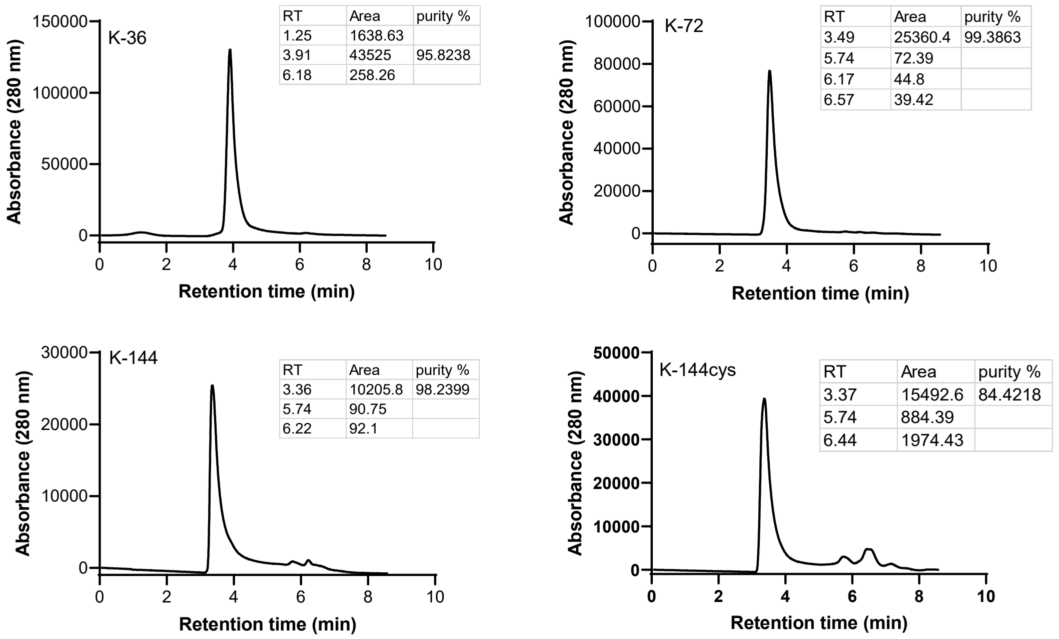

Supplementary Figure 6 HPLC analysis of K-proteins tested at 2.0 mg/mL. All K-proteins (K-36, K-72, K-144, and K-144cys) were eluted at around 3.5-4.0 min in the HPLC chromatograph. The purity of K-proteins was determined by integration the peaks, showing excellent purity: 95.8% for K-36, 99.4% for K-72, 98.2% for K-144, and 88.4% for K-144cys.

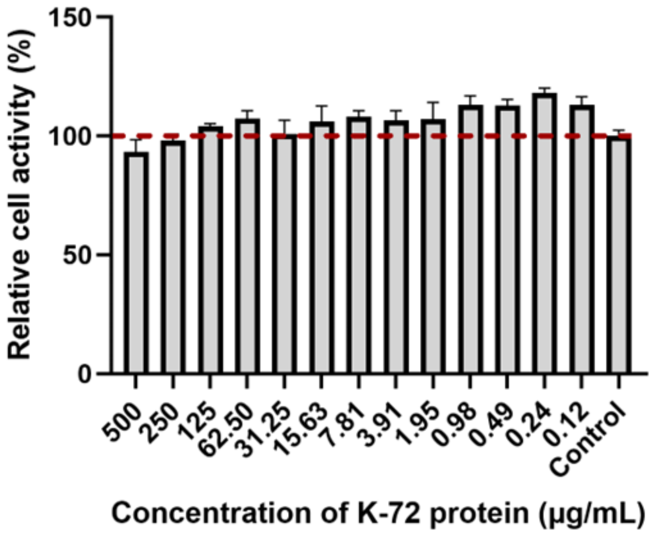

Supplementary Figure 7 Cytotoxicity tests of K-72 proteins with a concentration ranging from 0.12-500 μg·mL^{-1}. Owing to its high molecular weight, the cell viability is still above 88% when the concentration of K-protein is as high as 500 μg·mL^{-1}, showing low cytotoxicity of K-proteins.

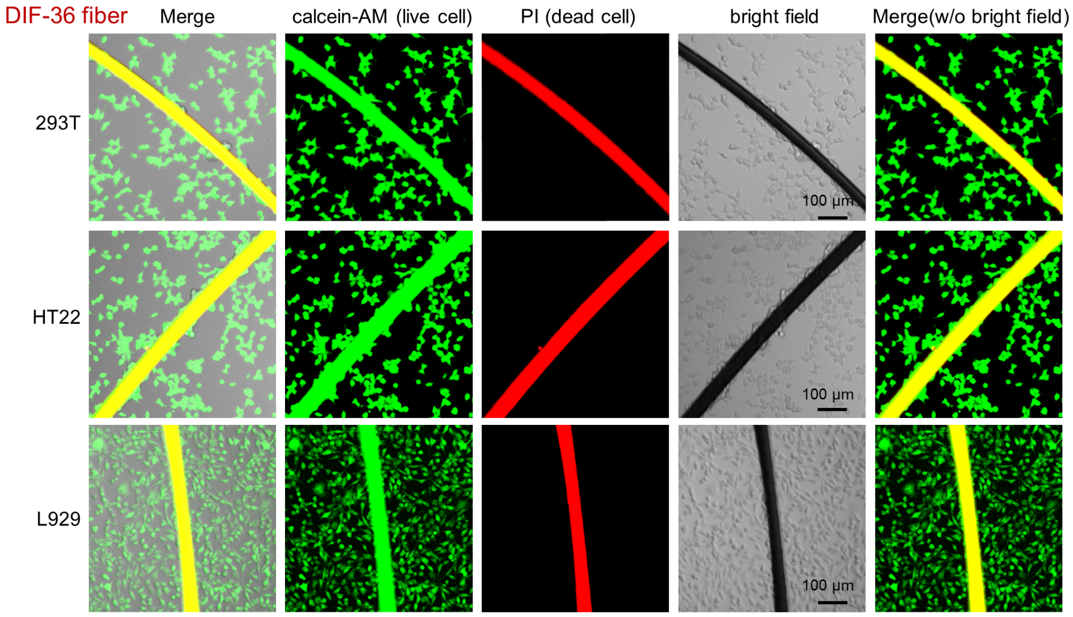

Supplementary Figure 8 Cytotoxicity tests of the DIF-36 fibers. The DIF-36 fibers were co-cultured with HEK293T, L929, or HT22 cells. If not specified, live cells are stained with calcein-AM and dead cells are stained with propidium iodide (PI). Dead cells were barely observed and the DIF-36 fibers exhibited no toxicity to co-cultured cells, even for cells adhered on the fiber surfaces.

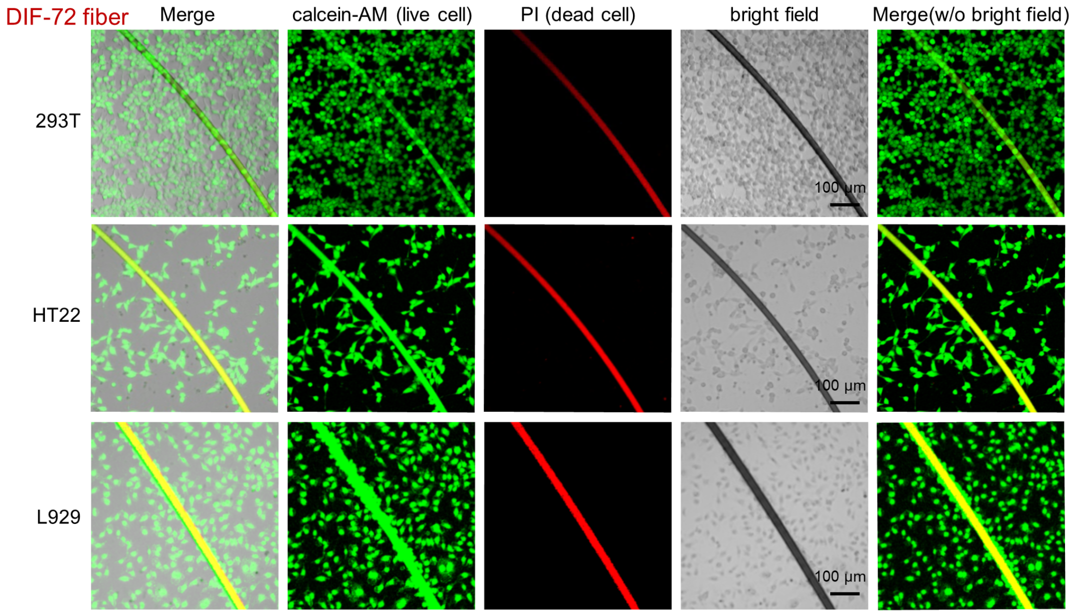

Supplementary Figure 9 Cytotoxicity tests of the DIF-72 fibers. The DIF-72 fibers were co-cultured with HEK293T, L929, or HT22 cells. Dead cells were barely observed and the DIF-72 fibers exhibited no toxicity to co-cultured cells, even for cells adhered on the fiber surfaces.

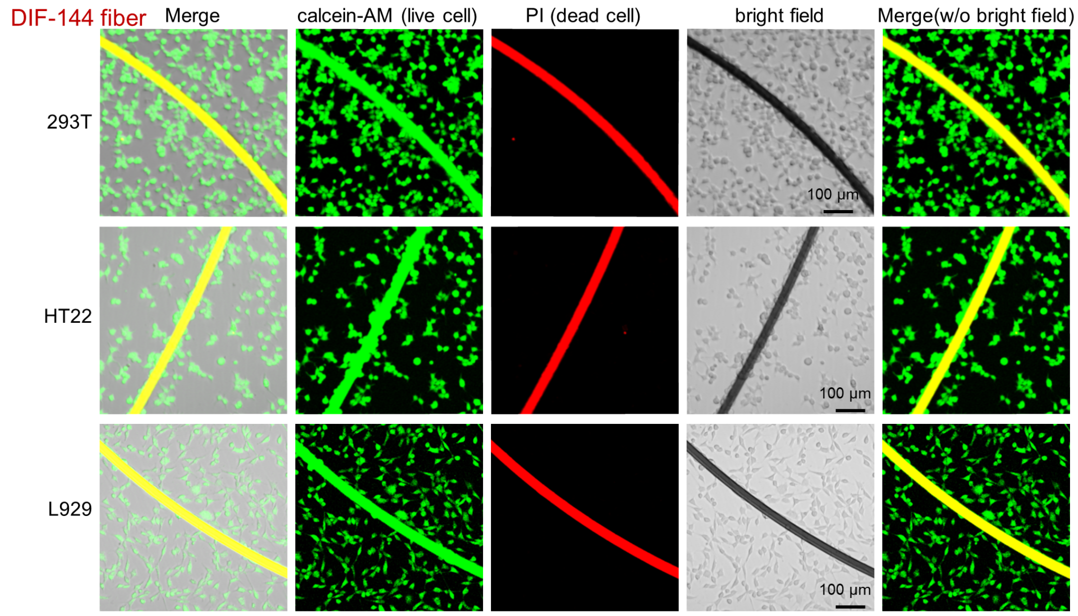

Supplementary Figure 10 Cytotoxicity tests of the DIF-144 fibers. The DIF-144 fibers were co-cultured with HEK293T, L929, or HT22 cells. Dead cells were barely observed and the DIF-144 fibers exhibited no toxicity to co-cultured cells, even for cells adhered on the fiber surfaces.

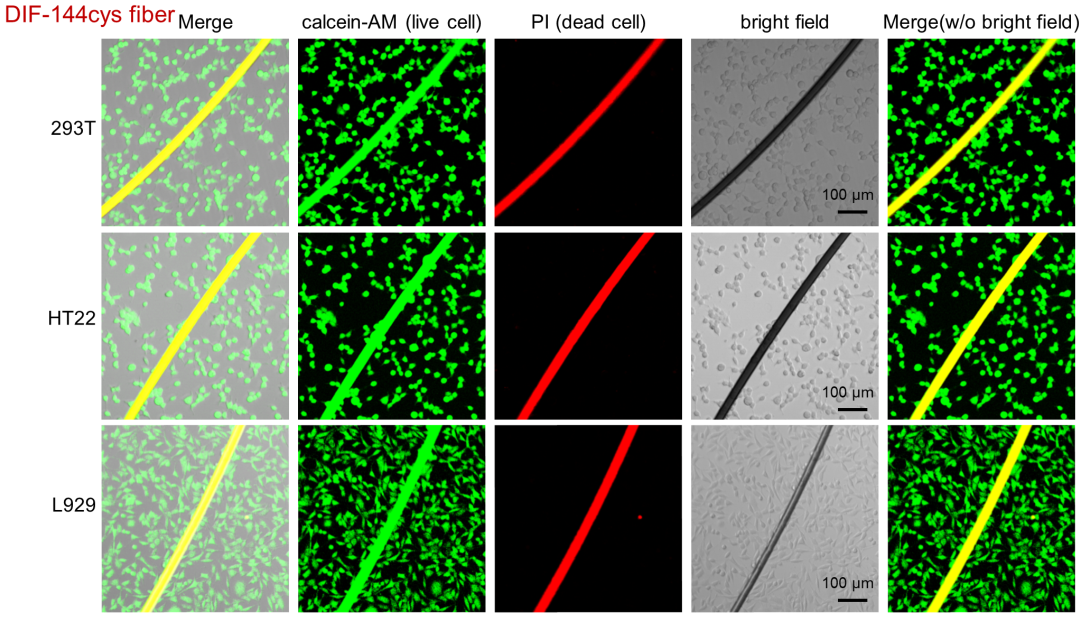

Supplementary Figure 11 Cytotoxicity tests of the DIF-144cys fibers. The DIF-144cys fibers were co-cultured with HEK293T, L929, or HT22 cells. Dead cells were barely observed and the DIF-144cys fibers exhibited no toxicity to co-cultured cells, even for cells adhered on the fiber surfaces.

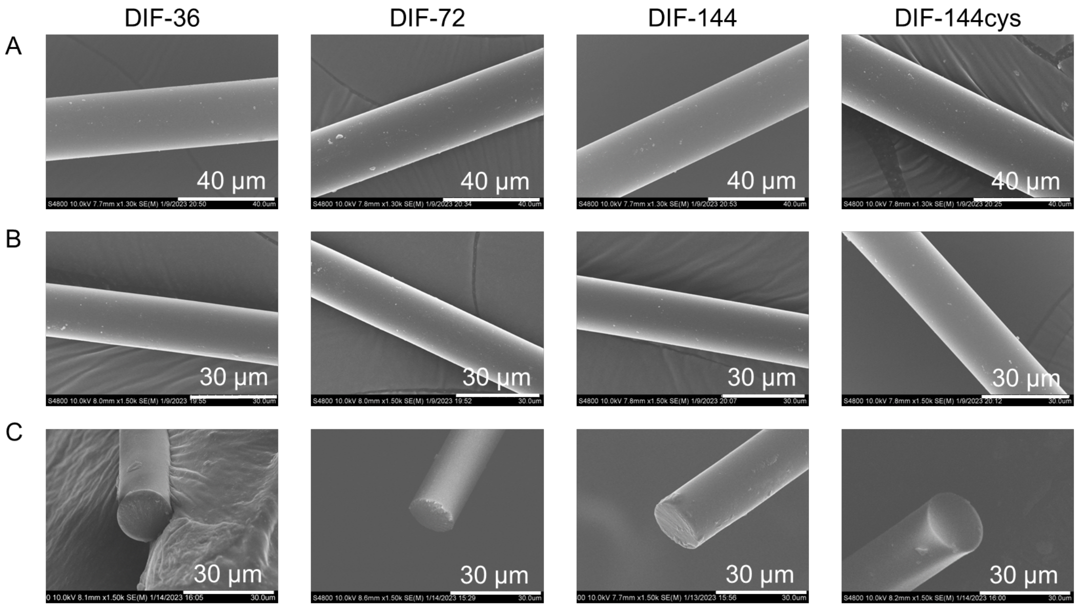

Supplementary Figure 12 SEM images of the DIF fibers before and after stretching. SEM images of (**A**) as-spun and (**B**) post-stretched DIF-36, DIF-72, DIF-144, and DIF-144cys fibers show cylindrical shapes and smooth surfaces. (**C**) Ruptured cross sections of the DIF-36, DIF-72, DIF-144, and DIF-144cys fibers show solid and flawless morphologies. The as-spun DIFs, post-stretched DIFs, and their fractured cross-sections all show similar morphology independent of their molecular weight.

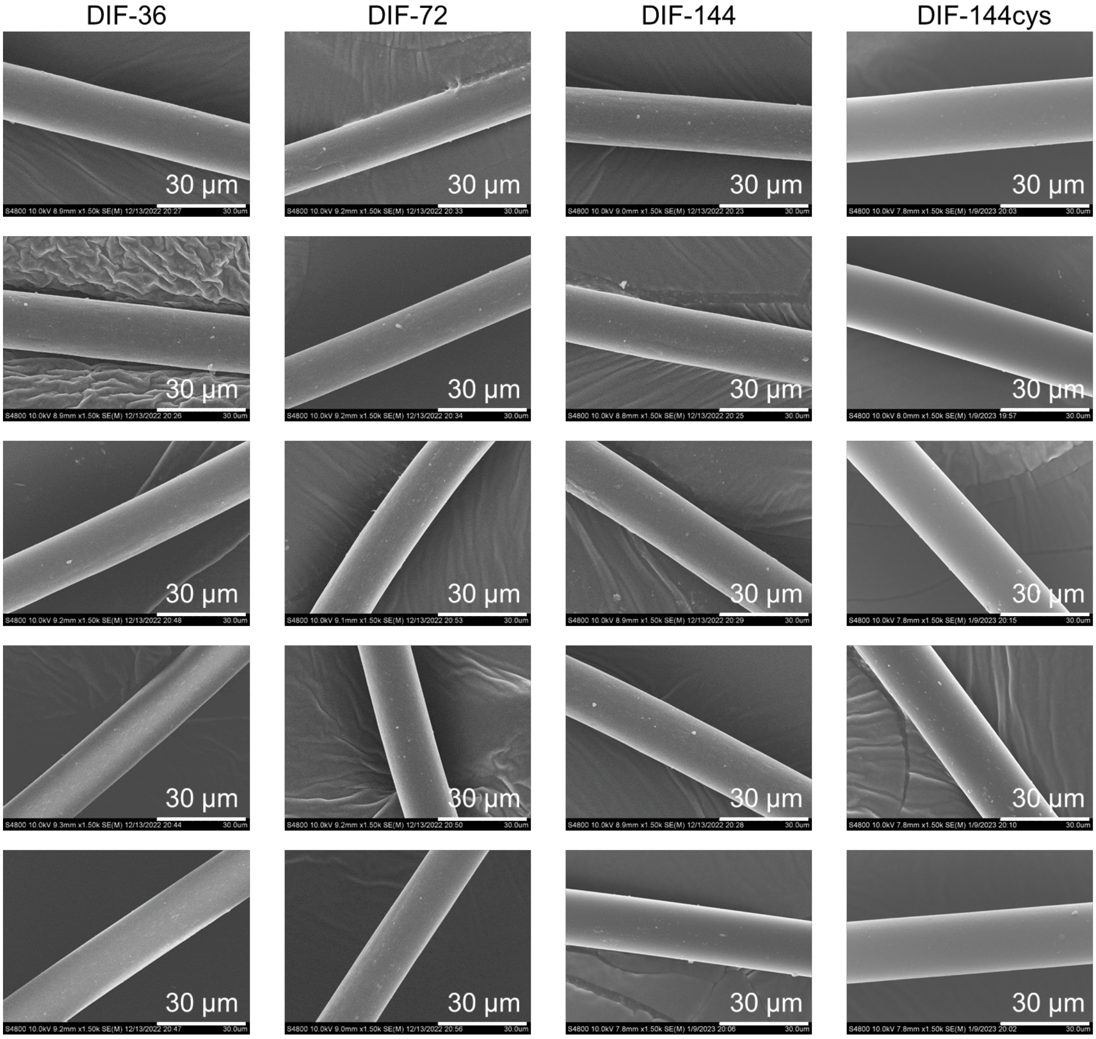

Supplementary Figure 13 SEM images for each type of the post-stretched fibers demonstrate the uniformity of the fibers. Scale bar: 30 μm.

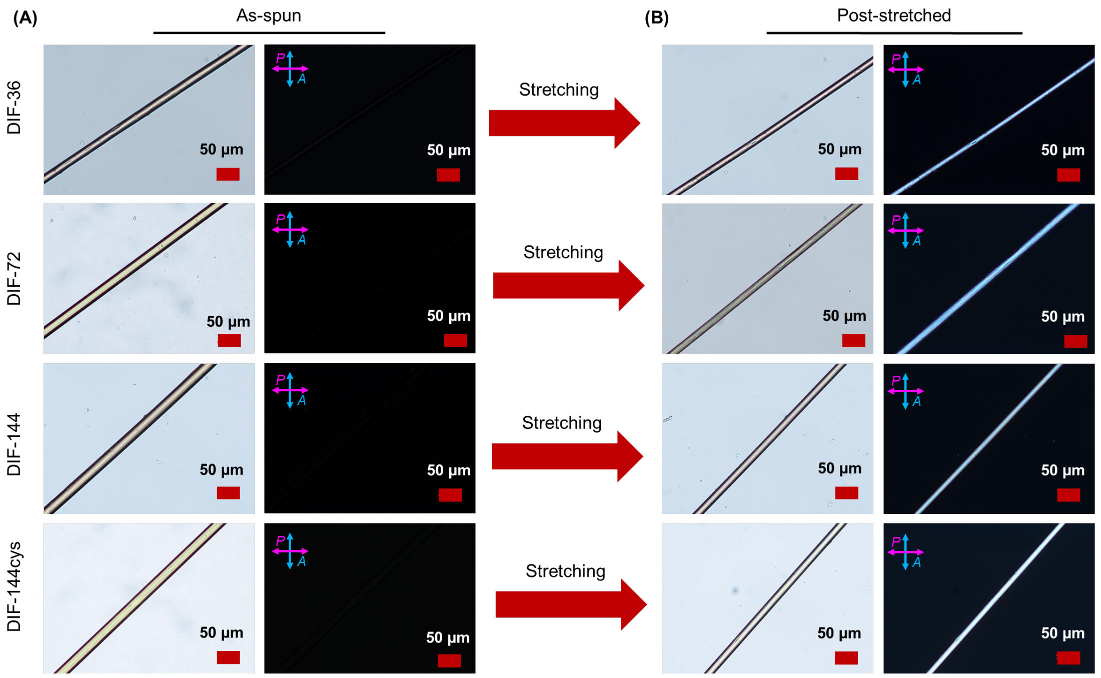

Supplementary Figure 14 Microscope images of the as-spun and post-stretched DIF fibers without and with crossed polarizers. No birefringence is observed in the as-spun DIF fibers under crossed polarizers, while a clear birefringence is observed in the post-stretched DIF fibers, suggesting orientational order in the post-stretched fibers.

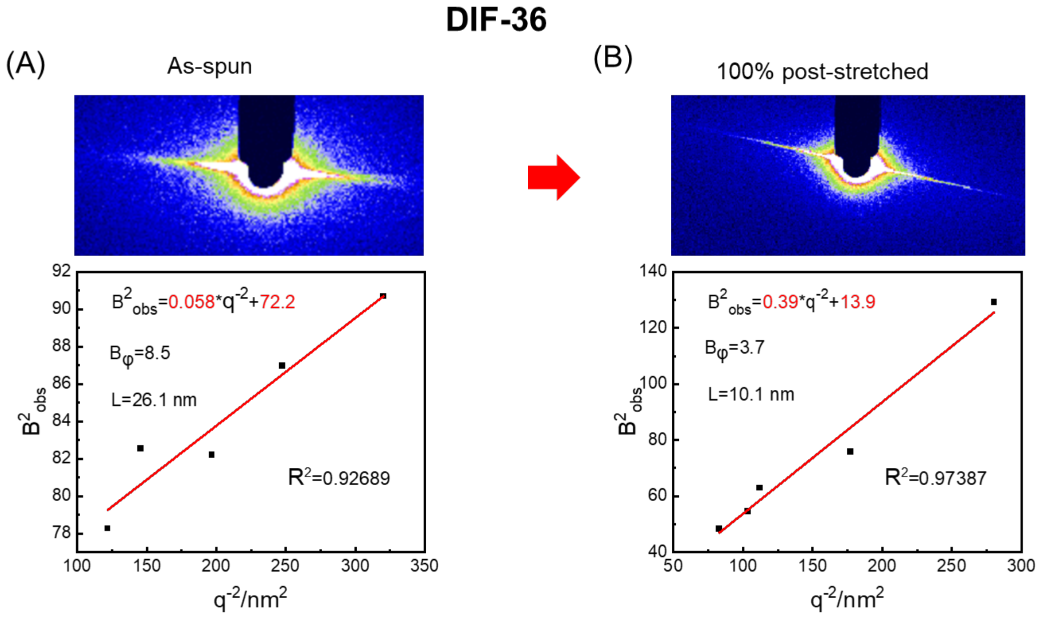

Supplementary Figure 15 2D SAXS patterns and B^2_{obs} versus q^{-2} diagrams of (**A**) as-spun and (**B**) post-stretched DIF-36 fibers. The post-stretched fibers show sharper streaks than the as-spun fibers, suggesting a more ordered molecular arrangement in the post-stretched fibers. In addition, the analysis of B^2_{obs} versus q^{-2} diagrams suggests that the periodic ordering distance decreases from 26.1 nm in the as-spun DIF-36 fibers to 10.1 nm in the post-stretched DIF-36 fibers.

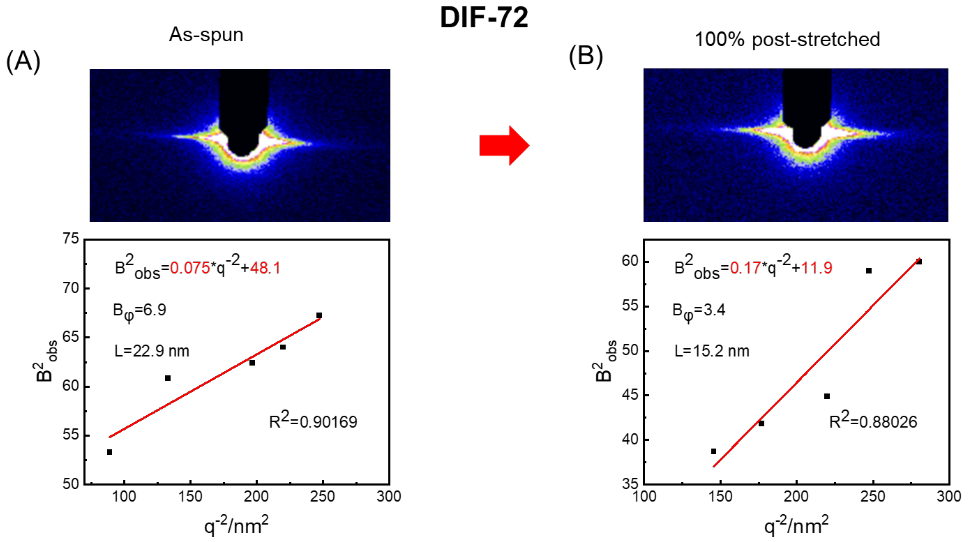

Supplementary Figure 16 2D SAXS patterns and B^2_{obs} versus q^{-2} diagrams of (**A**) as-spun and (**B**) post-stretched DIF-72 fibers. The post-stretched fibers show sharper streaks than the as-spun fibers, suggesting a more ordered molecular arrangement in the post-stretched fibers. In addition, the analysis of B^2_{obs} versus q^{-2} diagrams suggests that the periodic ordering distance decreases from 22.9 nm in the as-spun DIF-72 fibers to 15.2 nm in the post-stretched DIF-72 fibers.

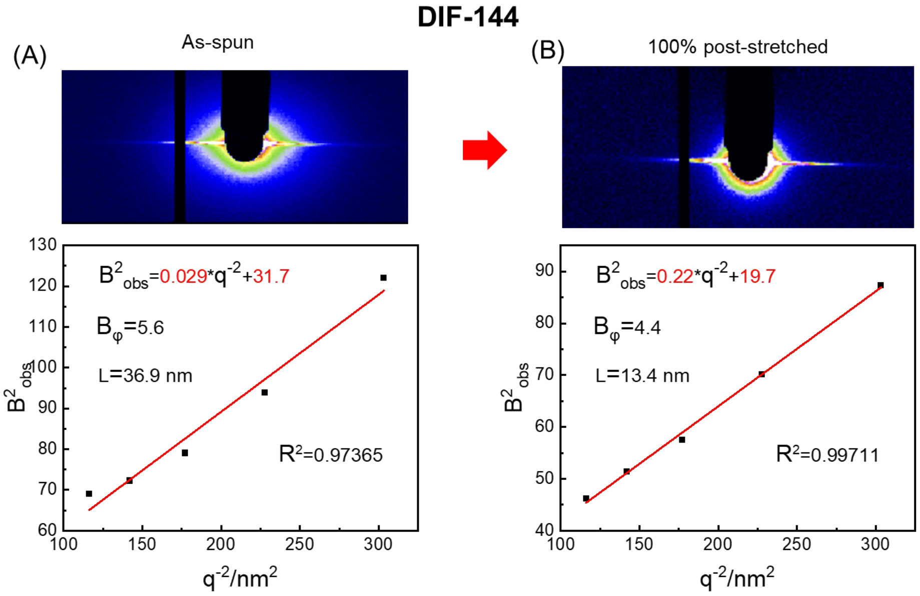

Supplementary Figure 17 2D SAXS patterns and B^2_{obs} versus q^{-2} diagrams of (**A**) as-spun and (**B**) post-stretched DIF-144 fibers. The post-stretched fibers show sharper streaks than the as-spun fibers, suggesting a more ordered molecular arrangement in the post-stretched fibers. In addition, the analysis of B^2_{obs} versus q^{-2} diagrams suggests that the periodic ordering distance decreases from 36.9 nm in the as-spun DIF-144 fibers to 13.4 nm in the post-stretched DIF-144 fibers.

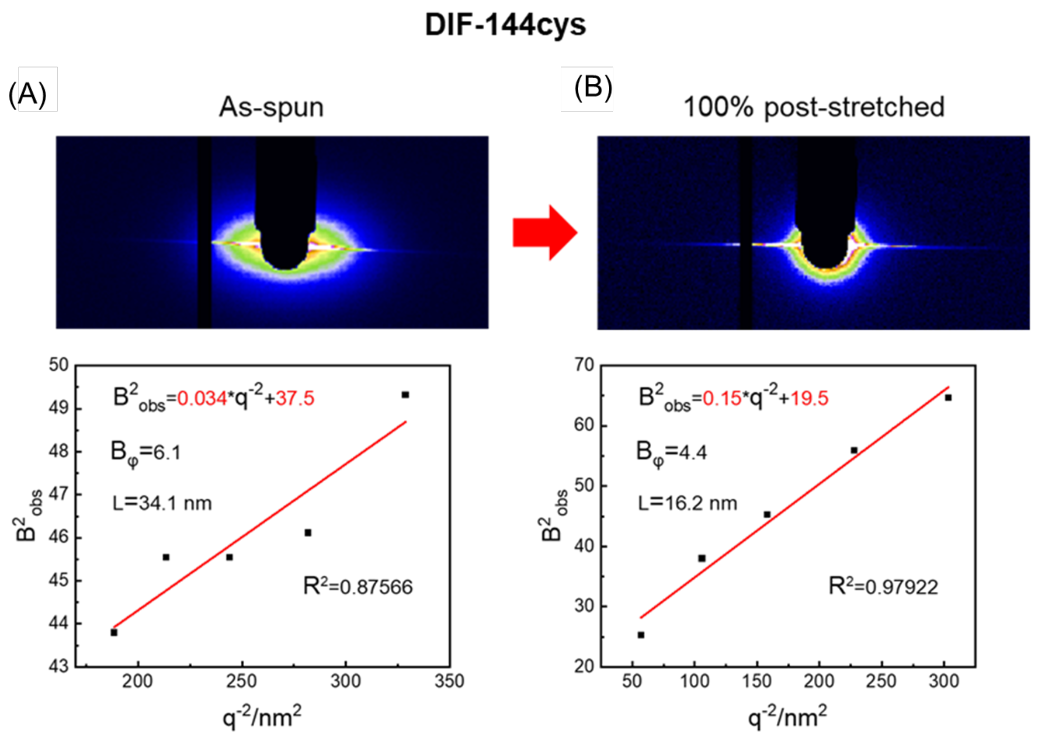

Supplementary Figure 18 2D SAXS patterns and B^2_{obs} versus q^{-2} diagrams of (**A**) as-spun and (**B**) post-stretched DIF-144cys fiber. The post-stretched fibers show sharper streaks than the as-spun fibers, suggesting a more ordered molecular arrangement in the post-stretched fibers. In addition, the analysis of B^2_{obs} versus q^{-2} diagrams suggests that the periodic ordering distance decreases from 34.1 nm in the as-spun DIF-144cys fibers to 16.2 nm in the post-stretched DIF-144cys fibers.

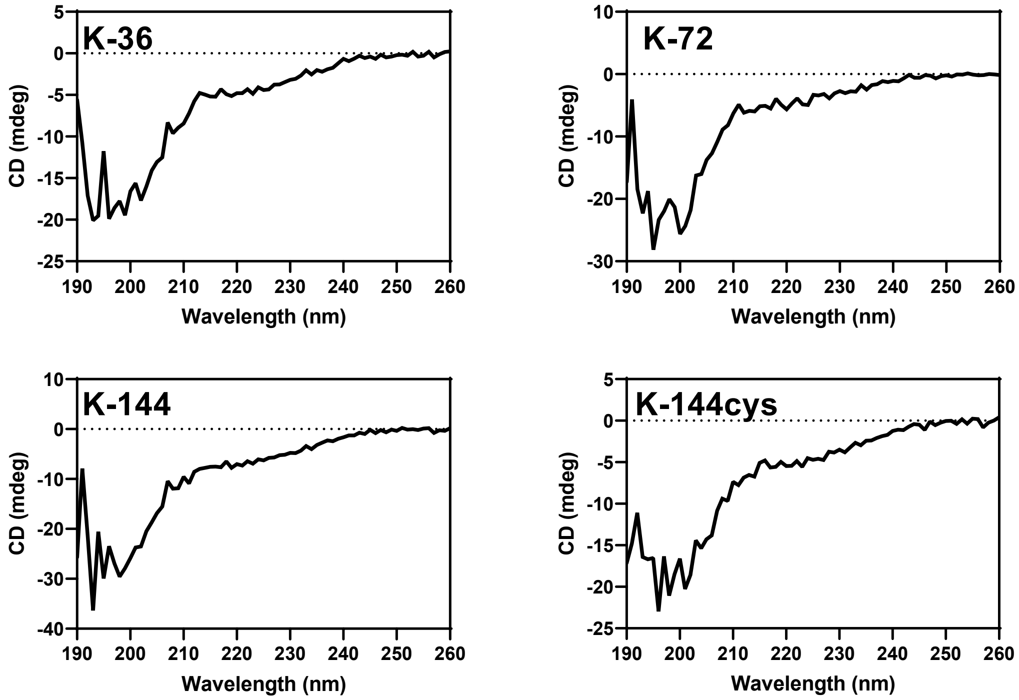

Supplementary Figure 19 CD spectra of different K-proteins (0.2 mg·mL^{-1} in H_2O). A negative signal was observed in the range of 190-200 nm for all K-proteins which indicates the presence of disordered, random coil structures within the K-proteins.

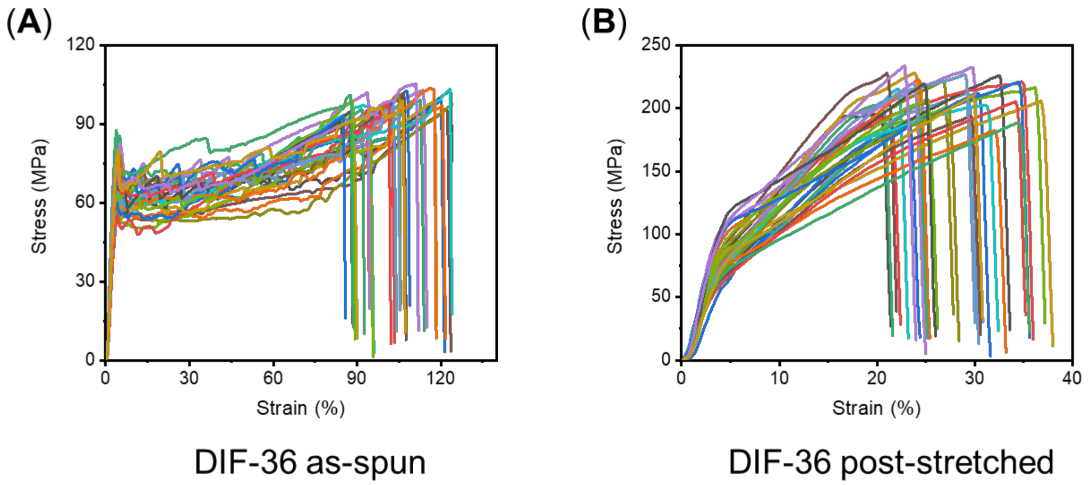

Supplementary Figure 20 Mechanical behaviors of the DIF-36 fibers. Stress-strain curves of **(A)** as-spun and **(B)** post-stretched DIF-36 fibers. n =30 samples.

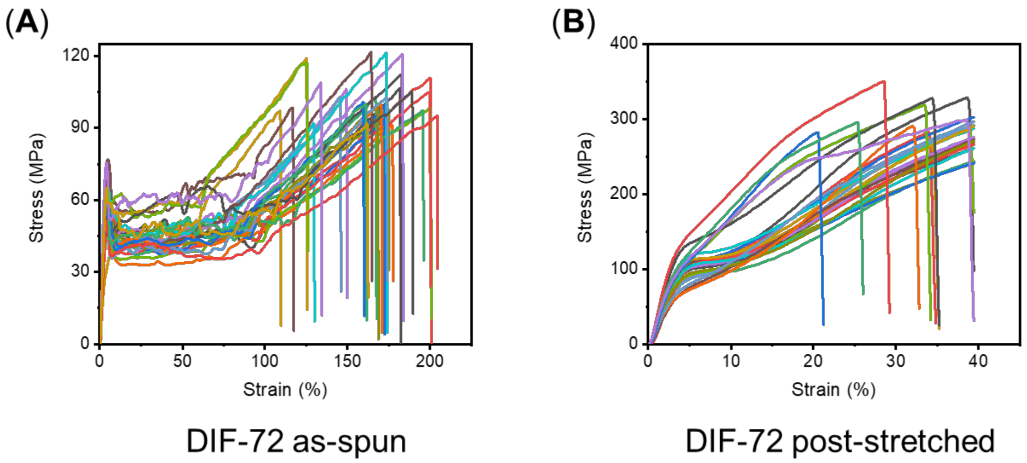

Supplementary Figure 21 Mechanical behaviors of the DIF-72 fibers. Stress-strain curves of **(A)** as-spun and **(B)** post-stretched DIF-72 fibers. n =30 samples.

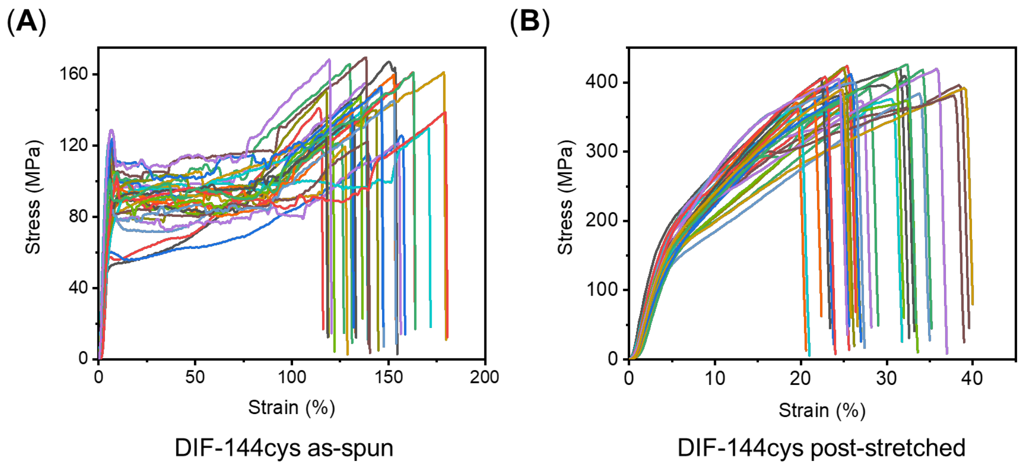

Supplementary Figure 22 Mechanical behaviors of the DIF-144cys fibers. Stress-strain curves of **(A)** as-spun and **(B)** post-stretched DIF-144cys fibers. n =30 samples.

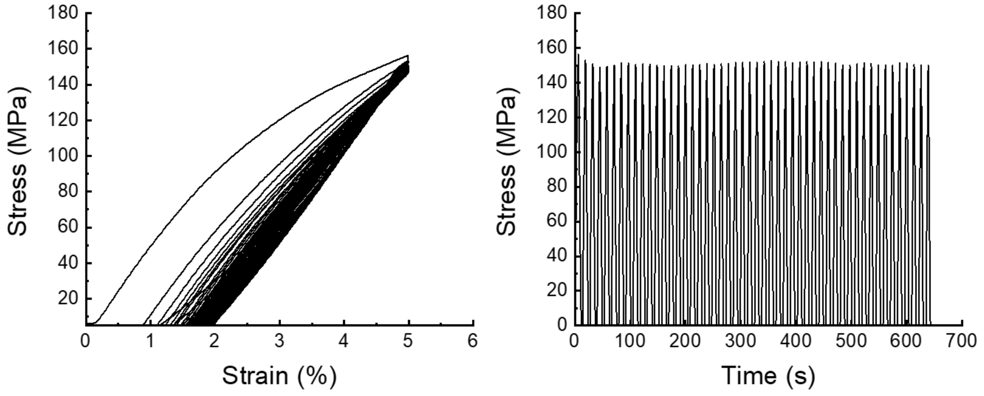

Supplementary Figure 23 Cyclic loading-unloading curves of the post-stretched DIF-72 fiber under 5% strain for 50 loops.

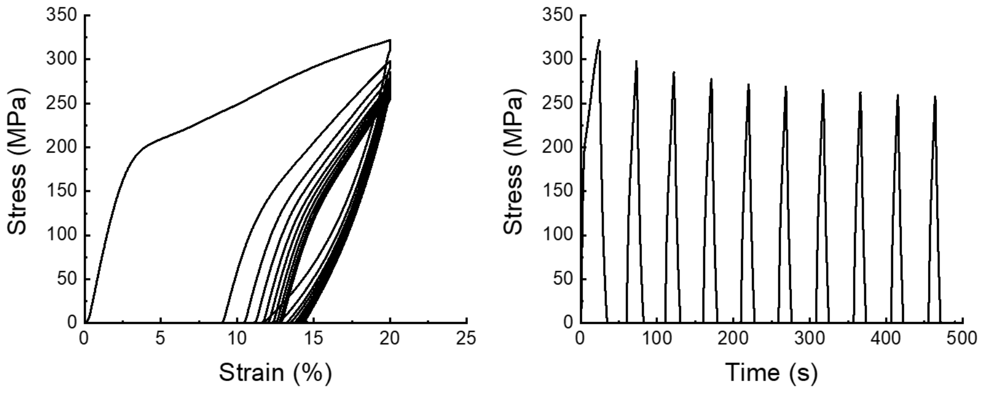

Supplementary Figure 24 Cyclic loading-unloading curves of the post-stretched DIF-72 fiber under 20% strain for 10 loops.

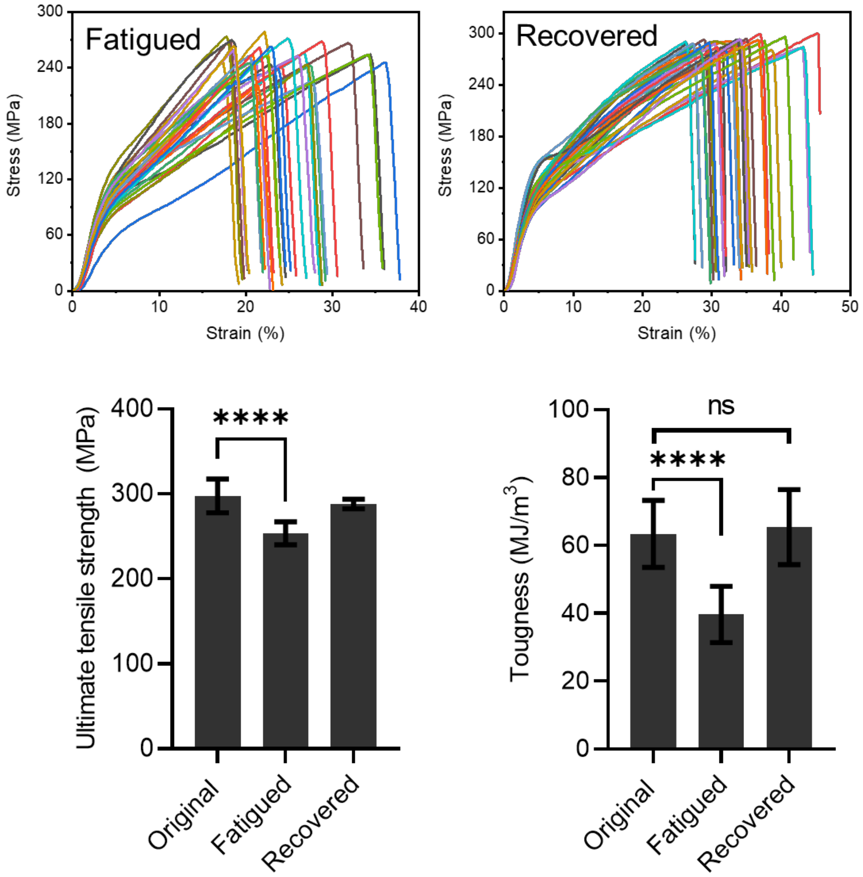

Supplementary Figure 25 Mechanical performance and stress-strain curves of fatigued and recovered DIF-72 fibers. After 2000 cyclic loading and unloading, the ultimate tensile strength and toughness of fatigued fibers is weakened to 253.6 ± 13.5 MPa and 39.7 ± 8.3 MJ/m^3, respectively. After recovery in a pH=8 aqueous solution and re-dried in the air, the ultimate tensile strength and toughness recovers to 288.2 ± 5.5 MPa and 65.4 ± 11.0 MJ/m^3, respectively, which is close to the original value of 297.7 ± 19.8 MPa and 63.4 ± 9.8 MJ/m^3. **** $p = 2.52 \times 10^{-10} < 0.0001$ (ultimate tensile strength, original vs fatigued), **** $p = 5.39 \times 10^{-13} < 0.0001$ (Toughness, original vs fatigued), ns, no significant difference. n =30 samples. If not specified, the statistical analysis was performed with GraphPad Prism 8.0 software. The results are obtained by statistical approach of two-way t-tests in this study.

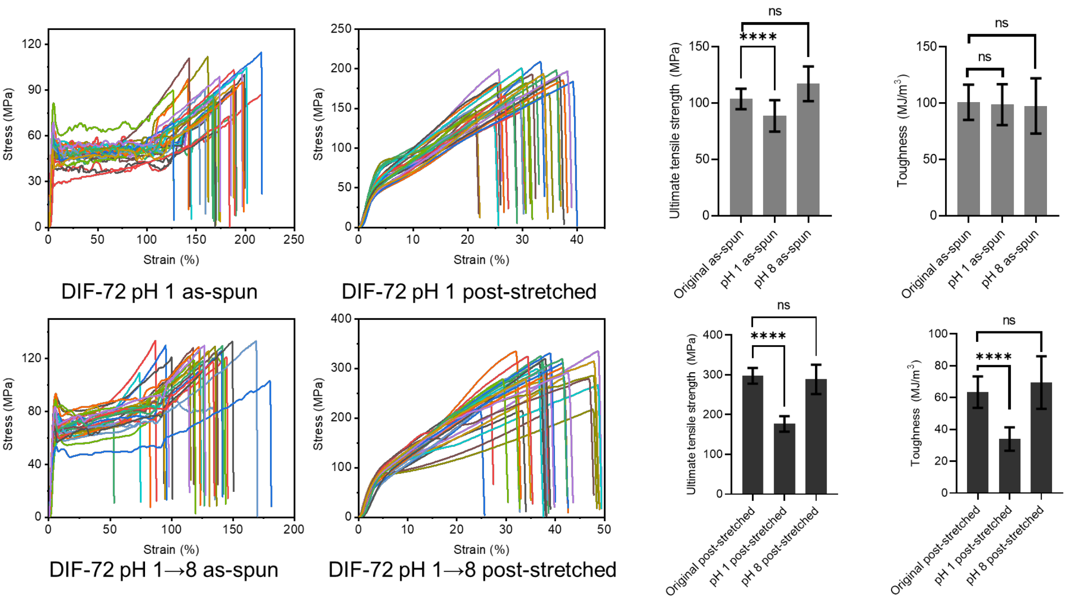

Supplementary Figure 26 Stress-strain curves and mechanical performance of the DIF-72 fibers immersed at pH=1 for 12 hours and recovered at pH=8 for 12 hours. After pH = 1 treatment, the ultimate tensile strength is weakened to 88.8 ± 13.9 MPa for the as-spun DIF-72 fibers, while the corresponding toughness values were in the range of 110 to 120 MJ/m^3 without any significant difference. For the post-stretched DIF-72 fibers, the ultimate tensile strength and toughness reduced to 176.7 ± 19.1 MPa and 34 ± 7.2 MJ/m^3, respectively. After recovery in a pH=8 aqueous solution and re-dried in the air, the ultimate tensile strength and toughness recovers to their original value for the DIF-72 fibers. **** $p = 0.000026 < 0.0001$ (Ultimate tensile strength for as-spun DIF fibers, pH= 1 vs original), **** $p = 1.09 \times 10^{-20} < 0.0001$ (Ultimate tensile strength for post-stretched DIF fibers, pH= 1 vs original), **** $p = 3.27 \times 10^{-14} < 0.0001$ (Toughness for post-stretched DIF fibers, pH= 1 vs original), ns. no significant difference. n =30 samples.

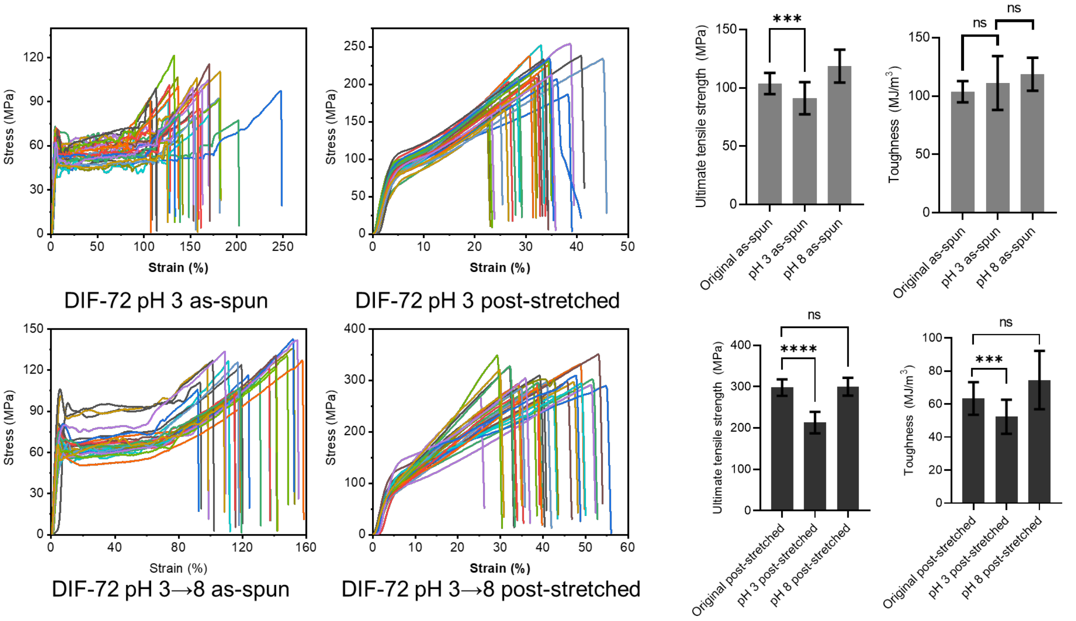

Supplementary Figure 27 Stress-strain curves and mechanical performance of the DIF-72

fibers immersed at pH=3 for 12 hours and recovered at pH=8 for 12 hours. After pH = 3 treatment, the ultimate tensile strength is weakened to 91.4 ± 14.1 MPa for the as-spun DIF-72 fibers, while the corresponding toughness values were in the range of 110 to 120 MJ/m^3 without any significant difference. For the post-stretched DIF-72 fibers, the ultimate tensile strength and toughness reduced to 213.3 ± 25.6 MPa and 52.3 ± 10.1 MJ/m^3, respectively. After recovery in a pH=8 aqueous solution and re-dried in the air, the ultimate tensile strength and toughness recovers to their original value for the DIF-72 fibers. *** $p = 0.0001 < 0.001$ (Ultimate tensile strength for as-spun DIF fibers, pH = 3 vs original), **** $p = 4.51 \times 10^{-15} < 0.0001$ (Ultimate tensile strength for post-stretched DIF fibers, pH = 3 vs original), *** $p = 0.00022 < 0.001$ (Toughness for post-stretched DIF fibers, pH= 3 vs original), ns. no significant difference. n =30 samples.

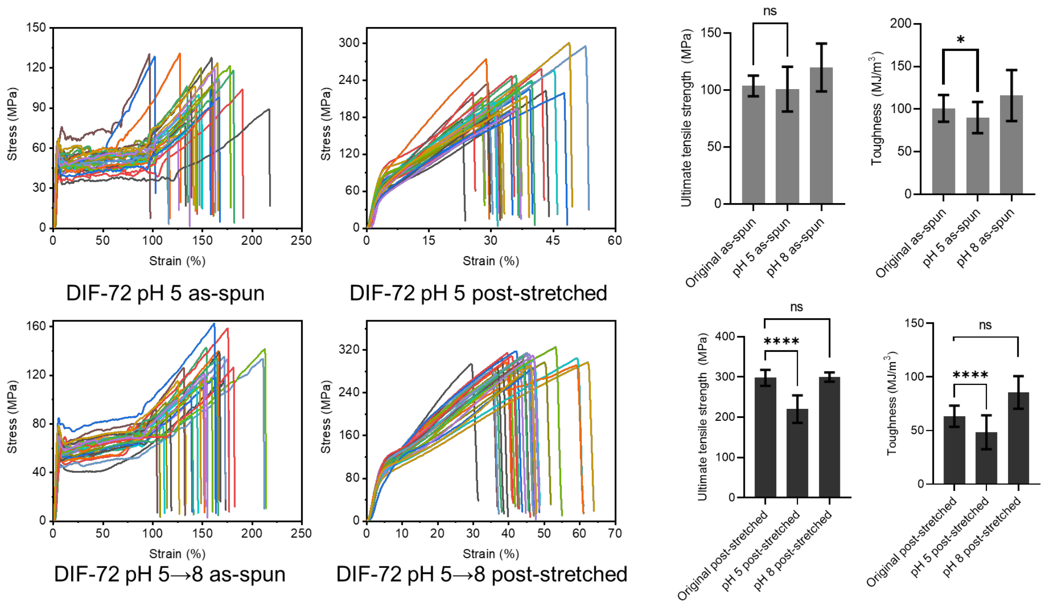

Supplementary Figure 28 Stress-strain curves and mechanical performance of the DIF-72 fibers immersed at pH=5 for 12 hours and recovered at pH=8 for 12 hours. After pH = 5 treatment, the ultimate tensile strength is weakened to 100.9 ± 19.3 MPa for the as-spun DIF-72 fibers, and the toughness is slightly decreased to 90.1 ± 17.9 MJ/m^3. For the post-stretched DIF-72 fibers, the ultimate tensile strength and toughness reduced to 220.2 ± 33.3 MPa and 48.5 ± 15.5 MJ/m^3, respectively. After recovery in a pH=8 aqueous solution and re-dried in the air, the ultimate tensile strength and toughness recovers to their original value for the DIF-72 fibers. * $p = 0.02 < 0.05$ (Toughness for as-spun DIF fibers, pH = 5 vs original), **** $p = 1.76 \times 10^{-11} < 0.0001$ (Ultimate tensile strength for post-stretched DIF fibers, pH = 5 vs original), **** $p = 4.31 \times 10^{-5} < 0.0001$ (Toughness for post-stretched DIF fibers, pH= 5 vs original), ns. no significant difference. n =30 samples.

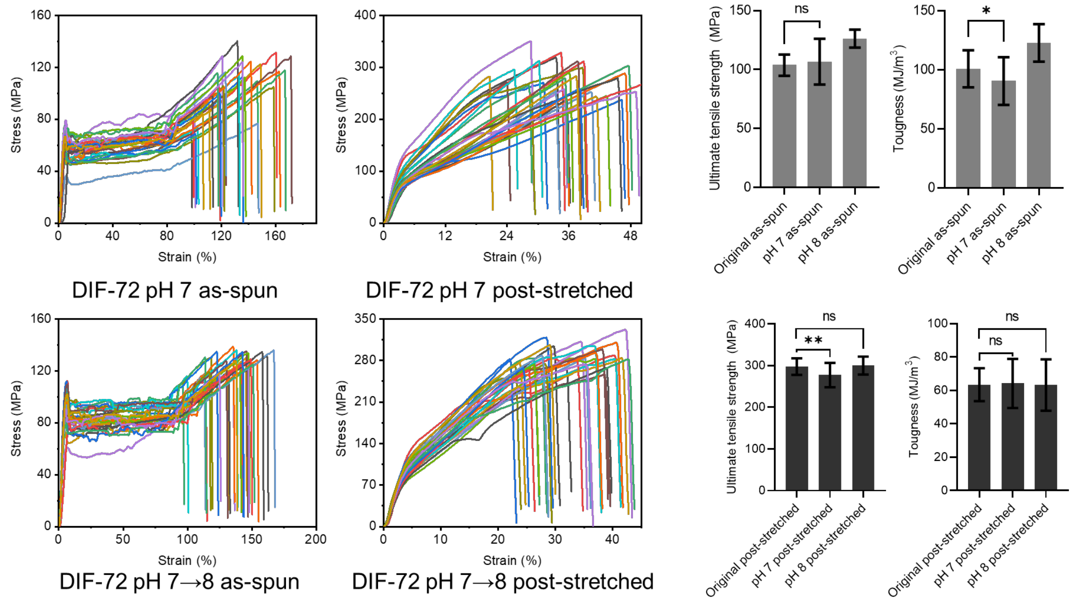

Supplementary Figure 29 Stress-strain curves and mechanical performance of the DIF-72 fibers immersed at pH=7 for 12 hours and recovered at pH=8 for 12 hours. After pH = 7 treatment, the ultimate tensile strength is 106.7 ± 19.5 MPa for the as-spun DIF-72 fibers, and the toughness is slightly decreased to 90.5 ± 20.1 MJ/m^3. For the post-stretched DIF-72 fibers, the ultimate tensile strength and toughness reduced to 277.2 ± 29.2 MPa and 64.2 ± 14.7 MJ/m^3, respectively. After recovery in a pH=8 aqueous solution and re-dried in the air, the ultimate tensile strength and toughness recovers to their original value for the DIF-72 fibers. * $p = 0.03 < 0.05$ (Toughness for as-spun DIF fibers, pH = 7 vs original), ** $p = 0.0024 < 0.01$ (Ultimate tensile strength for post-stretched DIF fibers, pH = 7 vs original), ns. no significant difference. n =30 samples.

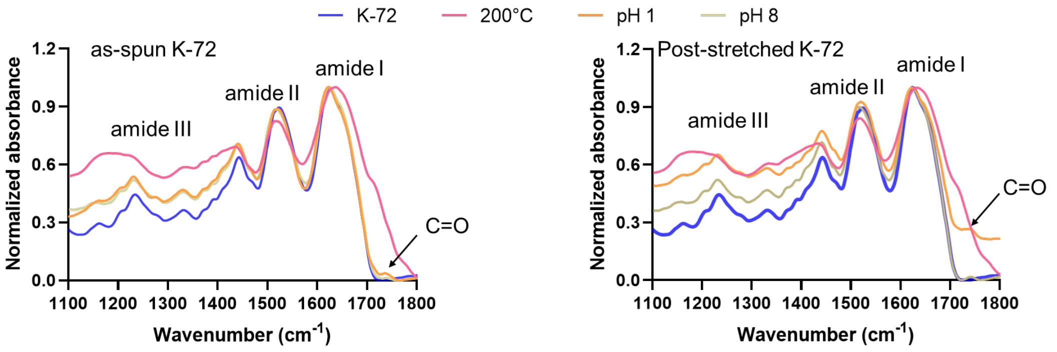

Supplementary Figure 30 The FT-IR analysis of K-72 protein and DIF-72 fibers at different conditions, including the variations of pH and high temperature (200°C). For the acid treatment, the stretching vibration of the C=N (imine) bond (1610-1640 cm^{-1}) was not observed, however, the stretching vibration of the C=O (aldehyde) from the hydrolyzed imine bonds for both as-spun and post-stretched DIF-72 fibers was detected at ~ 1740 cm^{-1}, indicating the acid-induced hydrolysis of imine bonds within the DIF fibers, thereby resulted in the decreased mechanical performance. At 200°C, the stretching vibration of the C=N bond was also not observed, and the stretching vibration

of amide bonds changed, especially for the amide III bands, indicating that the protein chain network has been partly and irreversibly broken, leading to a decrease in the ultimate tensile strength and toughness of the DIF fibers.

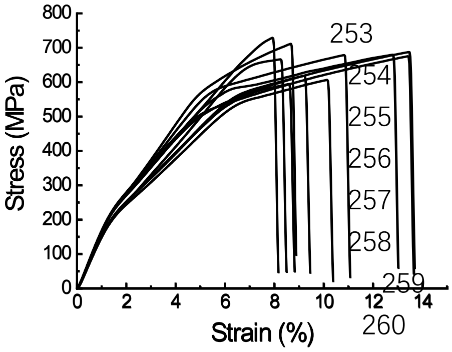

Supplementary Figure 31 Stress-strain curves of degummed natural silkworm fibers. Degummed natural silkworm silk fibers exhibit an ultimate tensile strength of 664 ± 42 MPa and a toughness of 47 ± 13 MJ/m^3. n =10 samples.

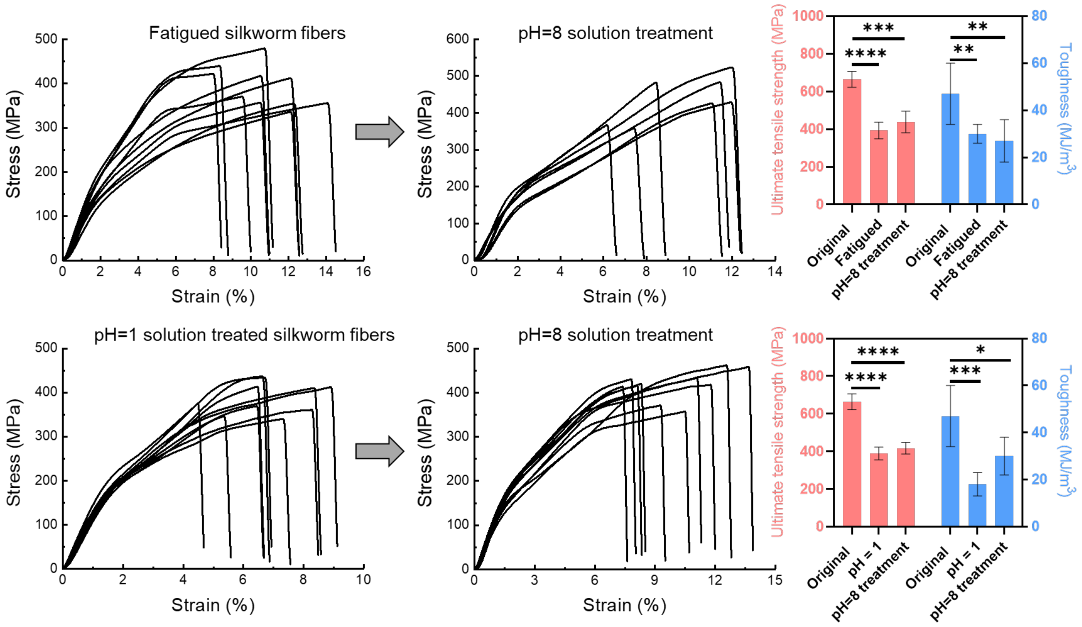

Supplementary Figure 32 Stress-strain curves of fatigued degummed natural silkworm fibers after 2000 loading-unloading cycles, fatigued silkworm fibers treated by pH=8 solution, silkworm fibers treated by pH=1 solution, and silkworm fibers treated by pH=1 solution and then pH=8 solution. The ultimate tensile strength and toughness of both fatigued and pH=1 solution-treated silkworm fibers decrease to ~390 MPa and ~30 MJ/m^3, respectively. Neither of fatigued and pH=1 solution-treated silkworm fibers could fully restore their mechanical performances after treatment in pH=8 solution. **** $p = 1.82 \times 10^{-8} < 0.0001$ (Ultimate tensile strength, fatigued vs original), *** $p = 0.000163 < 0.001$ (Ultimate tensile strength, pH = 8 vs original), ** $p = 0.0014 < 0.01$ (Toughness, fatigued vs original), ** $p = 0.0073 < 0.01$ (Toughness, pH = 8 vs original), **** $p = 2.61 \times 10^{-7} < 0.0001$ (Ultimate tensile strength, pH = 1 vs original), **** $p = 2.67 \times 10^{-8} < 0.0001$ (Ultimate tensile strength, pH = 1→8 vs original), *** $p = 0.00014 < 0.001$ (Toughness, pH = 1 vs original), * $p = 0.016 < 0.05$ (Toughness, pH = 1→8 vs original), n =7-10 samples.

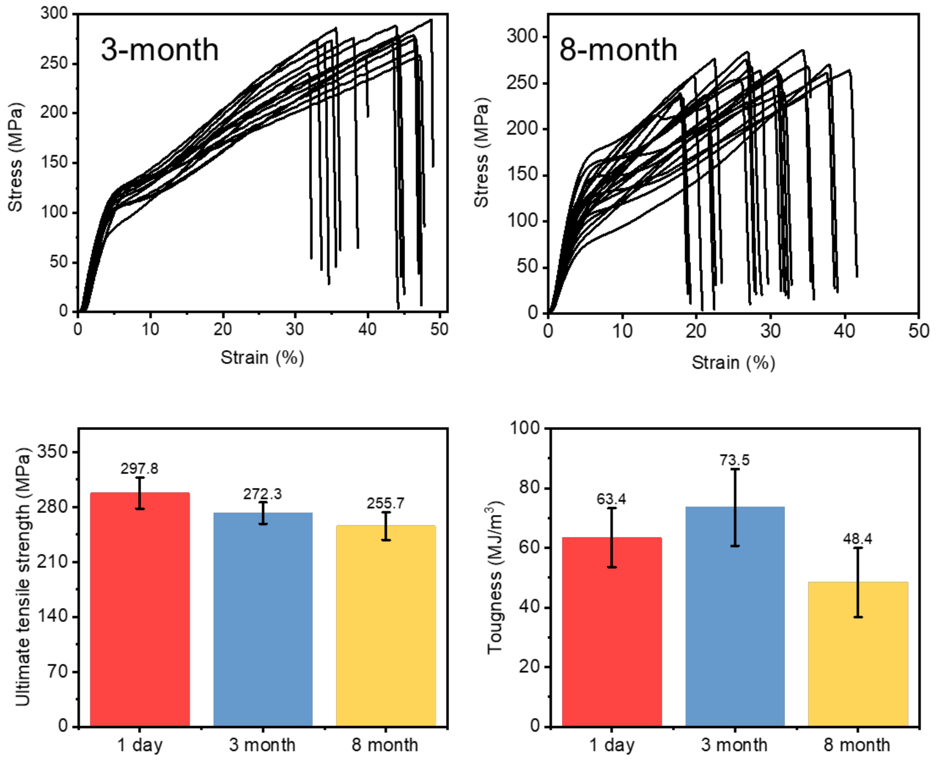

Supplementary Figure 33 Stability of the DIF-72 fibers after storage under ambient conditions (25°C, ~30% humidity, and nonsterile) for 3, and 8 months. The ultimate tensile strength and toughness of fresh-prepared DIF-72 fiber is ~298 MPa and ~ 63 MJ/m^3, respectively. After being stored under ambient conditions for 3, and 8 months, the ultimate tensile strength of DIF-72 fibers slightly decreased to 272 MPa and 256 MPa, respectively. While the toughness was in the range of 63 to 74 MJ/m^3. No distinguishable difference was observed in the mechanical performance of DIF-72 fibers after 3-, and 8-months' storage, suggesting the good stability of DIF-72 fibers.

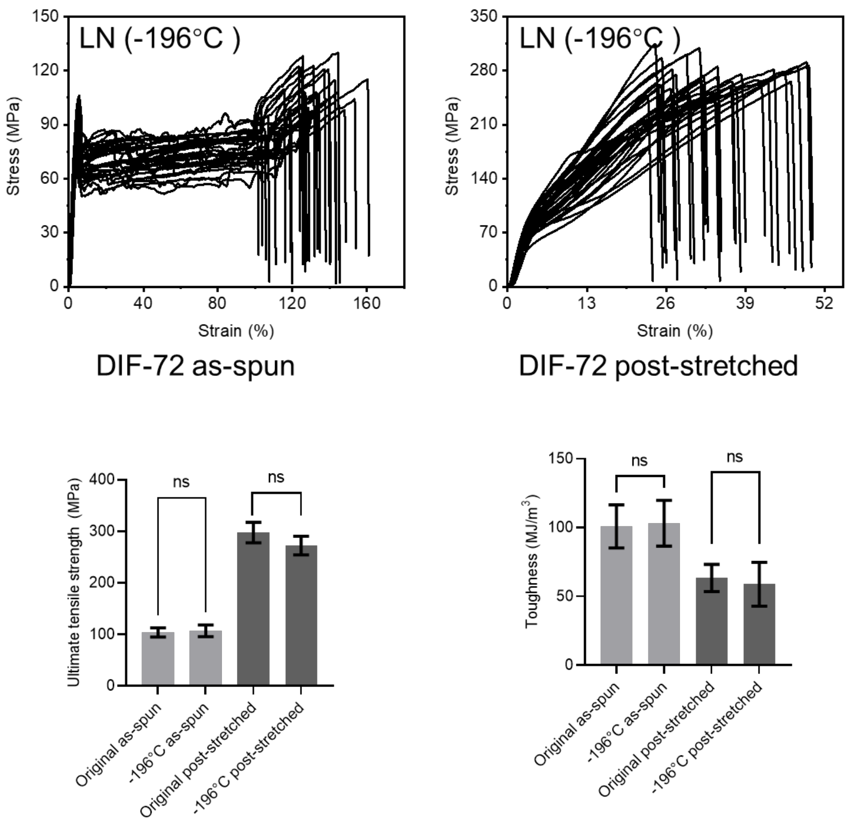

Supplementary Figure 34 Stress-strain curves of the as-spun and post-stretched DIF-72 fibers treated with liquid nitrogen for 12 hours and tested ex situ at room temperature (RT). The results showed no obvious decrease in the mechanical performances after low-temperature treatment. ns, no significant difference. n =30 samples. ns. no significant difference.

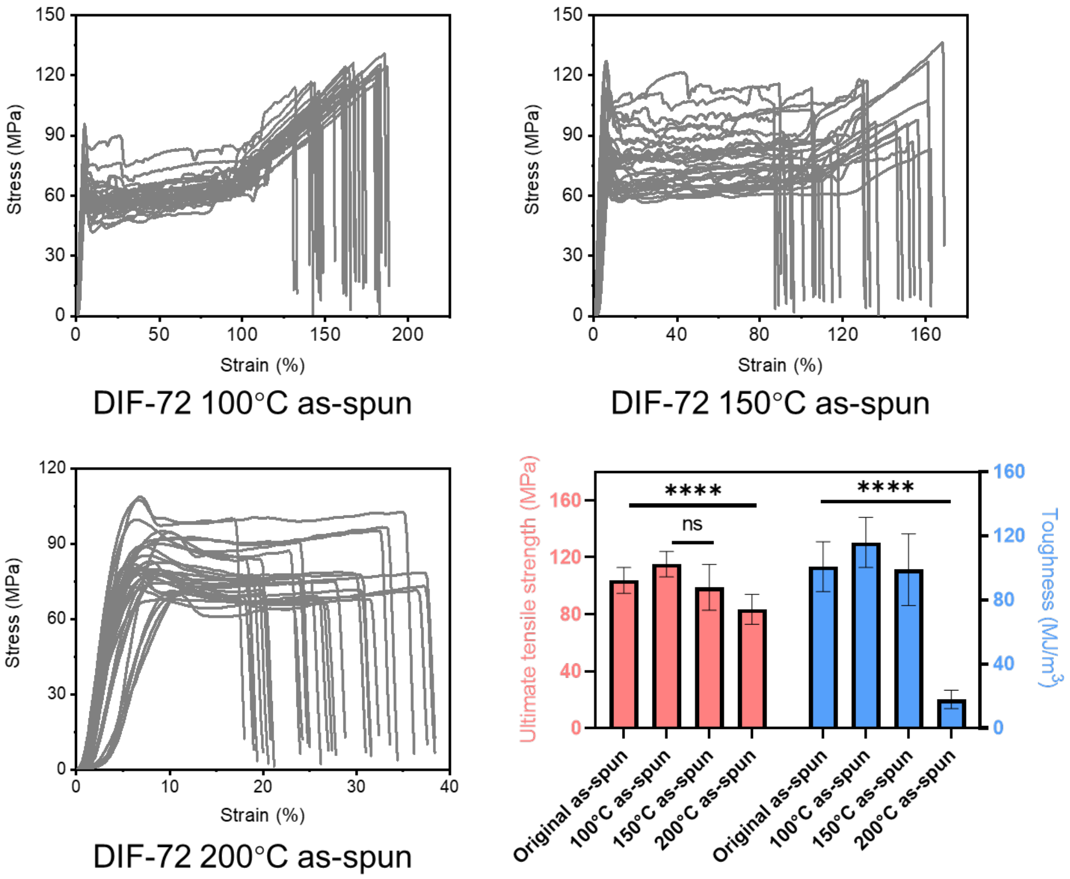

Supplementary Figure 35 Stress-strain curves of the as-spun fibers tested ex-situ at 100°C, 150°C, and 200°C, and tested at RT (room temperature). When the as-spun DIF-72 fibers were first treated at 100°C or 150°C for 12 hours and then tested ex-situ at RT, the ultimate tensile strength and toughness showed no significant difference. However, both values reduced significantly when further increasing temperature to 200°C, which cannot be recovered to its original values. **** $p = 1.96 \times 10^{-9} < 0.0001$ (Ultimate tensile strength for as-spun DIF fibers, 200°C vs original), **** $p = 3.56 \times 10^{-23} < 0.0001$ (Toughness for as-spun DIF fibers, 200°C vs original), ns, no significant difference. n =30 samples

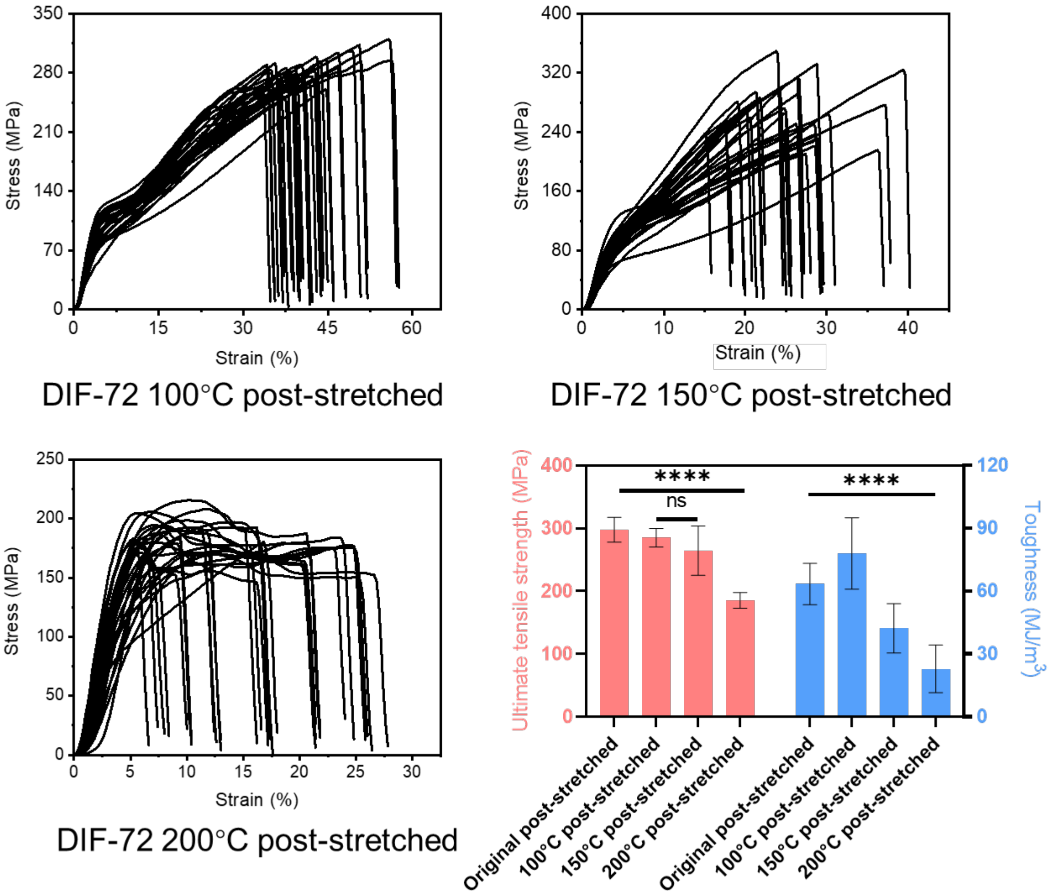

Supplementary Figure 36 Stress-strain curves of the post-stretched fibers tested *ex*-situ at 100°C, 150°C, and 200°C, and tested at RT (room temperature). When the post-stretched DIF-72 fibers were first treated at 100°C or 150°C for 12 hours and then tested ex-situ at RT, the ultimate tensile strength and toughness is comparable to the original DIF-72 fibers. In stark contrast, when further increasing temperature to 200°C, these values significantly decrease to 185.2 ± 12.5 MPa and 22.9 ± 11.3 MJ/m^3, respectively. **** $p = 3.91 \times 10^{-21} < 0.0001$ (Ultimate tensile strength for post-stretched DIF fibers, 200°C vs original), **** $p = 3.16 \times 10^{-15} < 0.0001$ (Toughness for post-stretched DIF fibers, 200°C vs original), ns, no significant difference. n =30 samples.

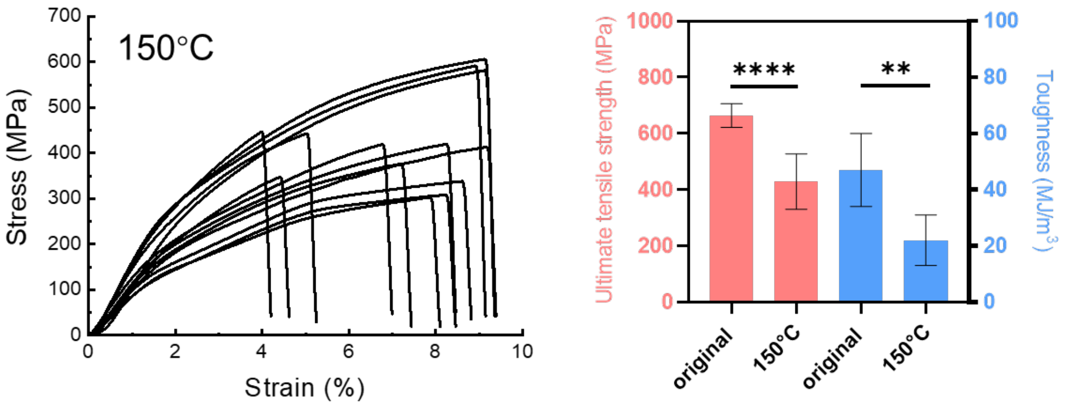

Supplementary Figure 37 Mechanical performances of degummed natural silkworm fibers

after heating to 150°C for 12 hours. The ultimate tensile strength and toughness of heated silk fibers irreversibly decrease to 429 ± 99 MPa and 22 ± 9 MJ/m^3, respectively. **** $p = 2.11 \times 10^{-5} < 0.0001$ (Ultimate tensile strength, 150°C vs original), ** $p = 0.0018 < 0.01$ (Toughness for, 150°C vs original), n = 13 samples.

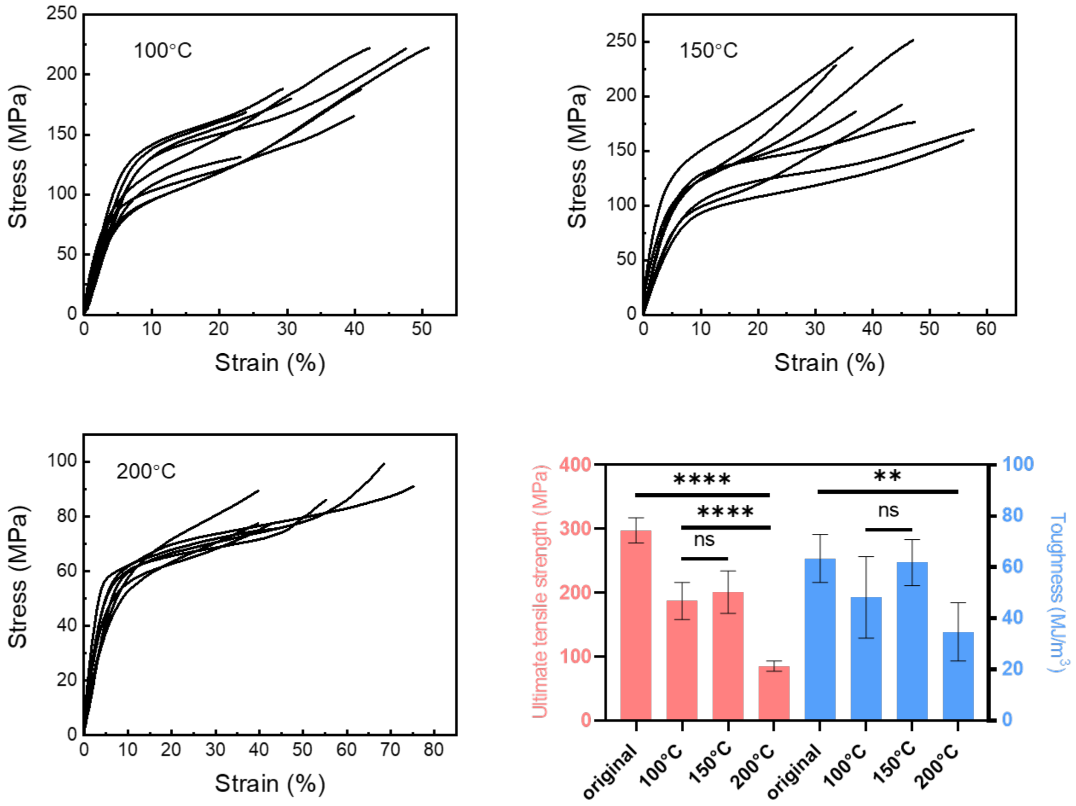

Supplementary Figure 38 In-situ tests of the post-stretched DIF-72 fibers at high temperatures (100°C, 150°C, and 200°C). The ultimate tensile strength and toughness of the DIF-72 fibers decrease to ~187 MPa, ~ 48 MJ/m^3 at 100°C, ~200 MPa, ~ 61 MJ/m^3 at 150°C, and ~85 MPa, ~ 35 MJ/m^3 at 200°C. **** $p = 1.90 \times 10^{-5} < 0.0001$ (Ultimate tensile strength for post-stretched DIF fibers, 200°C vs 100°C), **** $p = 7.51 \times 10^{-7} < 0.0001$ (Ultimate tensile strength for post-stretched DIF fibers, 200°C vs original), ** $p = 0.0022 < 0.01$ (Toughness for post-stretched DIF fibers, 200°C vs original), ns, no significant difference. Here we should mention that the in-situ testing is relatively time-consuming due to the complicated loading very thin single fiber in the DMA sample holder and the very slow heating and cooling process. It normally cost half day for every sample testing and only one DMA instrument can be shared in our institute. In this context, n = 7-9 samples for each group were tested.

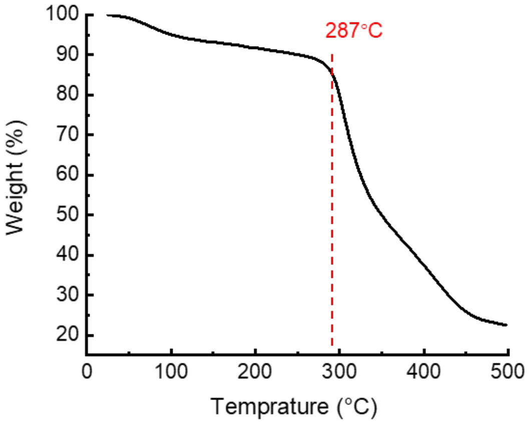

Supplementary Figure 39 Thermogravimetric analysis (TGA) of the DIF-72 fibers. The small decrease in weight below ~287°C may be attributed to the loss of water. The weight dramatically decreases above ~287°C due to the deposition of K-proteins, suggesting good thermal stability of K-proteins.

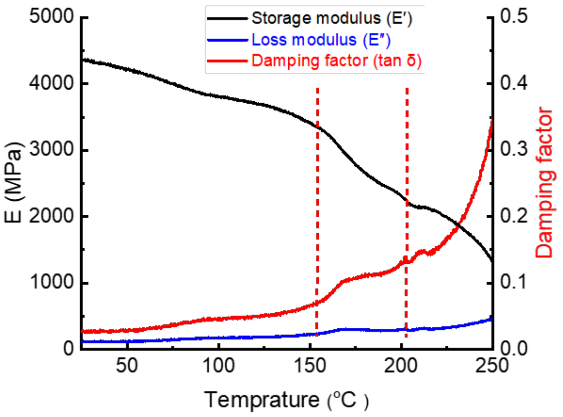

Supplementary Figure 40 Dynamic thermomechanical analysis (DMA) of the DIF-72 fibers. As the temperature increases, the storage modulus decreases while the loss modulus and damping factor increase.

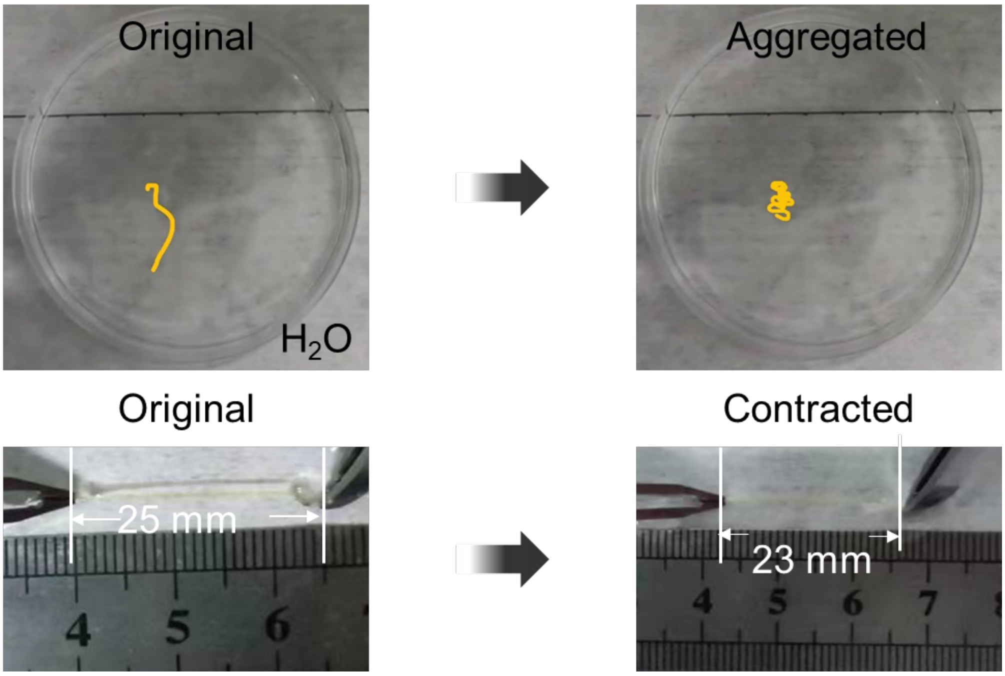

Supplementary Figure 41 Water-triggered contraction of the post-stretched DIF-72 fibers in water (pH = 8) over time. A yellow line highlights the fiber. Unlike the self-folding and extending of as-spun DIF-72 fibers in water over time, the post-stretched DIF-72 fibers exhibited rapid aggregation behavior when contacting water. The length of post-stretched DIF-72 fibers before hydration and after dehydration is 25 mm and 23 mm, respectively. This water-triggered contraction behavior is due to the relaxation of stretching-induced ordered protein chains to a less ordered state accompanied by an increase in entropy.

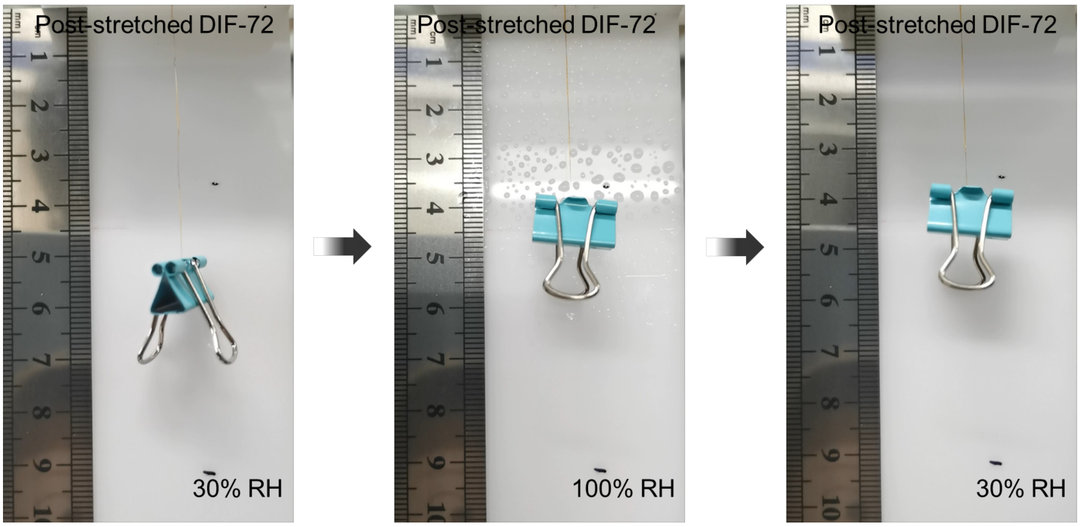

Supplementary Figure 42 Humidity-triggered contraction of the post-stretched DIF-72 fiber. It was found that the post-stretched DIF-72 fiber can only lift the clip by 25% of its original length when the fiber is hydrated at 100% humidity. However, when the post-stretched DIF fiber is dehydrated at 30% humidity, it cannot release the clip back to its original position.

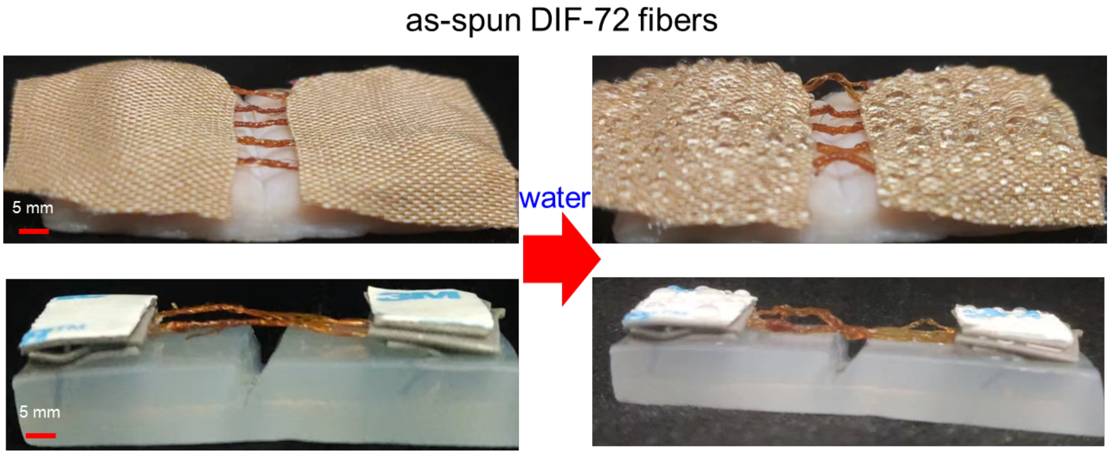

Supplementary Figure 43 Water-triggered extension of the as-spun DIF-72 fiber bundles on porcine skin (top) and agar gel (bottom). The results showed that the as-spun DIF-72 fiber bundles could not be applied to seal the notch in porcine skin or agar gel. When the fibers are hydrated, the imine bonds are partly hydrolyzed, and the as-spun DIF-72 fibers extend upon hydration, failing to seal the notch in porcine skin or agar gel. Scale bar: 5 mm.

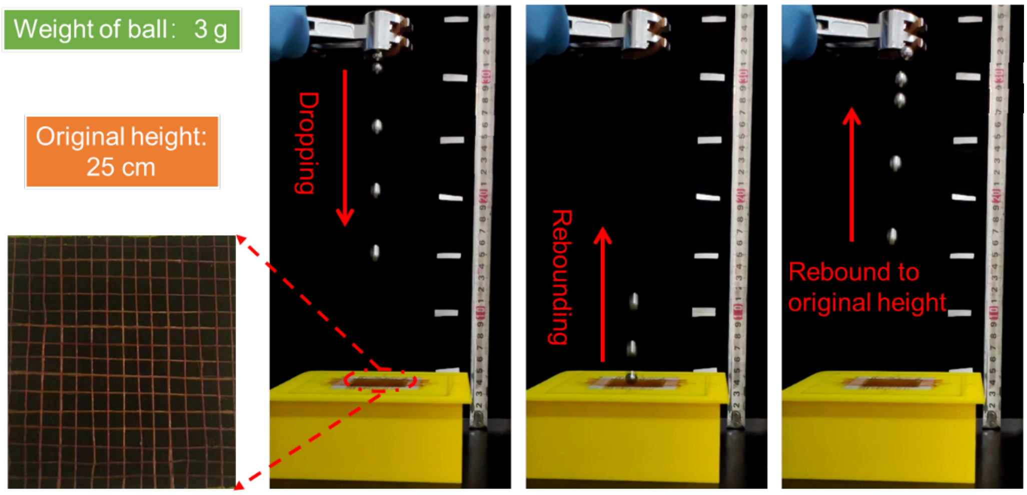

Supplementary Figure 44 Impact resistance of the DIF fibers. Rebounding of steal ball falling onto a DIF-72 fiber mesh. The steal ball essentially rebounds to its original height, suggesting an excellent impact resistance of DIF fibers.

Supplementary Movies

Movie 1 Self-folding and unfolding of the as-spun DIF-72 fibers in water at pH=8 over time.

Movie 2 Aggregation of the post-stretched DIF-72 fibers in water at pH=8 over time.

Movie 3 Reversible extension and contraction of the DIF-72 actuators triggered by humidity.

Movie 4 Contraction of the post-stretched DIF-72 fibers triggered by humidity.

Movie 5 Contraction of the post-stretched DIF-72 bundles upon hydration, sealing a 3 mm notch in agar gel.

Movie 6 Contraction of the post-stretched DIF-72 bundles upon hydration, sealing a 3 mm notch in porcine skin.

Movie 7 Water-triggered extension of the as-spun DIF-72 fiber bundles on agar gel.

Movie 8 Water-triggered extension of the as-spun DIF-72 fiber bundles on porcine skin.

Movie 9 Impact resistance of the DIF-72 fiber meshes.
