# Supplementary material for: Protein fibers with self-recoverable mechanical properties via dynamic imine chemistry
Source: Nat Commun. 2023 Sep 2;14:5348. doi: 10.1038/s41467-023-41084-1 (PMC10475138; doi:10.1038/s41467-023-41084-1)
Supplement: Supplementary file 2 — Reporting Summary [file 41467_2023_41084_MOESM2_ESM.pdf]

nature portfolio

Corresponding author(s): Kai Liu, Dong Chen, Juanjuan Su

Last updated by author(s): Aug 18, 2023

Reporting Summary

Nature Portfolio wishes to improve the reproducibility of the work that we publish. This form provides structure for consistency and transparency in reporting. For further information on Nature Portfolio policies, see our Editorial Policies and the Editorial Policy Checklist.

Statistics

For all statistical analyses, confirm that the following items are present in the figure legend, table legend, main text, or Methods section.

n/a	Confirmed	
☐	☒	The exact sample size (n) for each experimental group/condition, given as a discrete number and unit of measurement
☐	☒	A statement on whether measurements were taken from distinct samples or whether the same sample was measured repeatedly
☐	☒	The statistical test(s) used AND whether they are one- or two-sided<br>*Only common tests should be described solely by name; describe more complex techniques in the Methods section.*
☐	☒	A description of all covariates tested
☐	☒	A description of any assumptions or corrections, such as tests of normality and adjustment for multiple comparisons
☐	☒	A full description of the statistical parameters including central tendency (e.g. means) or other basic estimates (e.g. regression coefficient) AND variation (e.g. standard deviation) or associated estimates of uncertainty (e.g. confidence intervals)
☐	☒	For null hypothesis testing, the test statistic (e.g. F, t, r) with confidence intervals, effect sizes, degrees of freedom and P value noted<br>*Give P values as exact values whenever suitable.*
☒	☐	For Bayesian analysis, information on the choice of priors and Markov chain Monte Carlo settings
☒	☐	For hierarchical and complex designs, identification of the appropriate level for tests and full reporting of outcomes
☒	☐	Estimates of effect sizes (e.g. Cohen's d, Pearson's r), indicating how they were calculated

Our web collection on statistics for biologists contains articles on many of the points above.

Software and code

Policy information about availability of computer code

Data collection	N/A
Data analysis	The data analysis in this study was performed by Origin 2021, Graphpad Prism 8.0, and MestReNova 14.0.

For manuscripts utilizing custom algorithms or software that are central to the research but not yet described in published literature, software must be made available to editors and reviewers. We strongly encourage code deposition in a community repository (e.g. GitHub). See the Nature Portfolio guidelines for submitting code & software for further information.

Data

Policy information about availability of data

All manuscripts must include a data availability statement. This statement should provide the following information, where applicable:

- Accession codes, unique identifiers, or web links for publicly available datasets
- A description of any restrictions on data availability
- For clinical datasets or third party data, please ensure that the statement adheres to our policy

The authors confirm that the data supporting the findings of this study are available within the article (Figs. 1–5) and/or its supplementary materials (Supplementary Figures. 1–44, Tables 1–8, and Movies 1–9). Source data are provided in this paper. The tensile test, CD analysis, HPLC, DMA, TGA, SDS-PAGE, HPLC-TOF MS, cytotoxicity, SAXS data used in this study are provided in the Source Data file.

Human research participants

Policy information about studies involving human research participants and Sex and Gender in Research.

Reporting on sex and gender	N/A
Population characteristics	N/A
Recruitment	N/A
Ethics oversight	N/A

Note that full information on the approval of the study protocol must also be provided in the manuscript.

Field-specific reporting

Please select the one below that is the best fit for your research. If you are not sure, read the appropriate sections before making your selection.

- [x] Life sciences
- [ ] Behavioural & social sciences
- [ ] Ecological, evolutionary & environmental sciences

For a reference copy of the document with all sections, see nature.com/documents/nr-reporting-summary-flat.pdf

Life sciences study design

All studies must disclose on these points even when the disclosure is negative.

Sample size	The massive production of protein fibers (more than 50 cm in length) was achieved through wet-spinning technique. The preliminary experiment showed that DIF fibers have smooth morphologies. The mechanical performance of the resulting DIF fibers has low dispersion. Therefore 30 samples for each group were enough for the characterization. In addition, we should mention that the in-situ testing (DMA) is relatively time-consuming due to the complicated loading very thin single fiber in the DMA sample holder and the very slow heating and cooling process. It normally cost half day for every sample testing and only one DMA instrument can be shared in our institute. In this context, n = 7-9 samples for each group were tested.
Data exclusions	No data were excluded from the analyses.
Replication	The reproducibility of the experiments were confirmed with similar observations by the independent experiments and iterations with representatives.
Randomization	The fibers were prepared through wet-spinning technique and randomly divided into several different groups (pH, temperature, fatigue, and stability) before testing.
Blinding	All experiments and data collection was conducted as blinded including group allocation. All analyses were blinded to the experimental conditions.

Reporting for specific materials, systems and methods

We require information from authors about some types of materials, experimental systems and methods used in many studies. Here, indicate whether each material, system or method listed is relevant to your study. If you are not sure if a list item applies to your research, read the appropriate section before selecting a response.

Materials & experimental systems

n/a	Involved in the study	
x		Antibodies
	x	Eukaryotic cell lines
x		Palaeontology and archaeology
x		Animals and other organisms
x		Clinical data
x		Dual use research of concern

Methods

n/a	Involved in the study	
x		ChIP-seq
x		Flow cytometry
x		MRI-based neuroimaging

Eukaryotic cell lines

Policy information about cell lines and Sex and Gender in Research

Cell line source(s)	Human embryonic kidney cells 293T (catalog number:CL-0005, purchased from Procell Life Science & Technology, China), Mouse hippocampal neuronal cells HT22 (catalog number:CL-0697, purchased from Procell Life Science & Technology, China) Mouse fibroblast cells L929 (catalog number:CL-0137, purchased from Procell Life Science & Technology, China)
Authentication	Cell lines were authenticated by using the STR method
Mycoplasma contamination	All cell lines tested negative for mycoplasma contamination.
Commonly misidentified lines (See ICLAC register)	No commonly misidentified cell lines were used.
