# Supplementary material for: Protein fibers with self-recoverable mechanical properties via dynamic imine chemistry
Source: Nat Commun. 2023 Sep 2;14:5348. doi: 10.1038/s41467-023-41084-1 (PMC10475138; doi:10.1038/s41467-023-41084-1)
Supplement: Supplementary file 3 — Description of Additional Supplementary Files [file 41467_2023_41084_MOESM3_ESM.pdf]

Title: Supplementary Movie 1.

Description: Self-folding and unfolding of the as-spun DIF-72 fibers in water at pH=8 over time.

Title: Supplementary Movie 2.

Description: Aggregation of the post-stretched DIF-72 fibers in water at pH=8 over time.

Title: Supplementary Movie 3.

Description: Reversible extension and contraction of the DIF-72 actuators triggered by humidity.

Title: Supplementary Movie 4.

Description: Contraction of the post-stretched DIF-72 fibers triggered by humidity.

Title: Supplementary Movie 5.

Description: Contraction of the post-stretched DIF-72 bundles upon hydration, sealing a 3 mm notch in agar gel.

Title: Supplementary Movie 6.

Description: Contraction of the post-stretched DIF-72 bundles upon hydration, sealing a 3 mm notch in porcine skin.

Title: Supplementary Movie 7.

Description: Water-triggered extension of the as-spun DIF-72 fiber bundles on agar gel.

Title: Supplementary Movie 8.

Description: Water-triggered extension of the as-spun DIF-72 fiber bundles on porcine skin.

Title: Supplementary Movie 9.

Description: Impact resistance of the DIF-72 fiber meshes
